# Bibliometric analysis of congenital diaphragmatic hernia research models: evolution and insights from the past three decades

**DOI:** 10.1007/s00383-026-06597-y

**Published:** 2026-08-19

**Authors:** Boshen Shu, Xiaohui Wang, Richard Wagner, Florian Friedmacher, Martin Lacher, Ophelia Aubert

**Affiliations:** 1 Department of Pediatric Surgery, Henan Provincial People’s Hospital, Zhengzhou, Henan Province China; 2 Department of Pediatric Surgery, University of Leipzig, 04103 Leipzig, Germany; 3 Department of Pediatric Surgery, University Hospital Frankfurt, Goethe University Frankfurt, Frankfurt, Germany; 4 Department of Pediatric Surgery, University Medical Center Mannheim, University of Heidelberg, Theodor-Kutzer-Ufer 1-3, 68167 Mannheim, Germany

**Keywords:** CDH, Bibliometrics, Model, Citations, Visualized analysis

## Abstract

**Background:**

Research models of congenital diaphragmatic hernia (CDH) have evolved over time. We aimed to evaluate global research activities in CDH models using bibliometric analysis over the past three decades.

**Methods:**

Studies were retrieved from the Web of Science Core Collection (WoSCC) database between 1995 and 2024. Microsoft Excel, GraphPad Prism, and VOSviewer were applied to collect and analyze the bibliometric variables of this field.

**Results:**

A total of 302 original publications from 21 countries were identified. The nitrofen rat model (*n* = 187) was used in most studies, followed by sheep (*n* = 44), and rabbit (*n* = 32) models. The use of mouse models increased over time. The USA leads with the highest number of publications (*n* = 78) and collaborations, and the *Journal of Pediatric Surgery* published most articles. Main publication topics were developmental biology, pulmonary hypertension and surgical techniques. “Congenital diaphragmatic hernia”, “Nitrofen”, and “Pulmonary hypoplasia” were the most frequent keywords, while "Sildenafil", "Mice", and "Endothelial" were recent research hotspots.

**Conclusion:**

CDH research model has developed rapidly with increased collaborations and model diversity. Areas of interest varied, with a focus on developmental biology. Mouse models may gain importance to investigate genetic and molecular mechanisms of CDH.

**Supplementary Information:**

The online version contains supplementary material available at 10.1007/s00383-026-06597-y.

## Introduction

Congenital diaphragmatic hernia (CDH) is a relatively common birth defect, characterized by incomplete formation and/or muscularization of the diaphragm [1–4]. The pathobiology of CDH is still poorly understood but various genetic and environmental factors have been implicated [1, 2, 5–8]. The current incidence of CDH lies between 1.9 to 2.3 per 10,000 births [9, 10]. Even though neonatal intensive care has significantly improved over the past decades, mortality and long-term morbidity continue to be high [11, 12]. This is primarily attributed to persistent pulmonary hypoplasia and severe pulmonary hypertension, which involves complex pulmonary vascular remodeling and endothelial dysfunction as recently summarized in updated CDH translational research [11, 13, 14]. Reliable experimental models are essential tools for studying the underlying causes of CDH, as they can recapitulate the hallmarks of the disease and provide a platform for therapeutic testing. At present, a variety of CDH research models exist, including the nitrofen rat model, the sheep model and others [15]. Response to injury and therapeutic interventions may exhibit variability based on the model type and selecting an appropriate CDH model can pose a substantial challenge for researchers. Thus, using appropriate models in CDH research remains a constant objective for numerous global research and clinical groups.

Bibliometrics is an emerging method to analyze different aspects of published studies, such as quantity, quality, distribution, impact, collaboration, and research gaps [16, 17]. Over the years, policymakers have increasingly relied on bibliometrics to evaluate the research performance of scholars or to determine their eligibility for funding [18, 19]. This method has been widely applied in various research areas to evaluate the corresponding research activities or identify potential future trends [20–24]. To the best of our knowledge, only a few bibliometric or scientometric analyses have been conducted on CDH, and none have specifically examined the use of CDH research models [25, 26]. Hence, we aimed to systematically assess the use and characteristics of CDH research models via bibliometric and visualized analysis, in order to better understand how CDH research is performed and has evolved over the past three decades.

## Methods

### Data source and search strategy

**Fig. 1:**
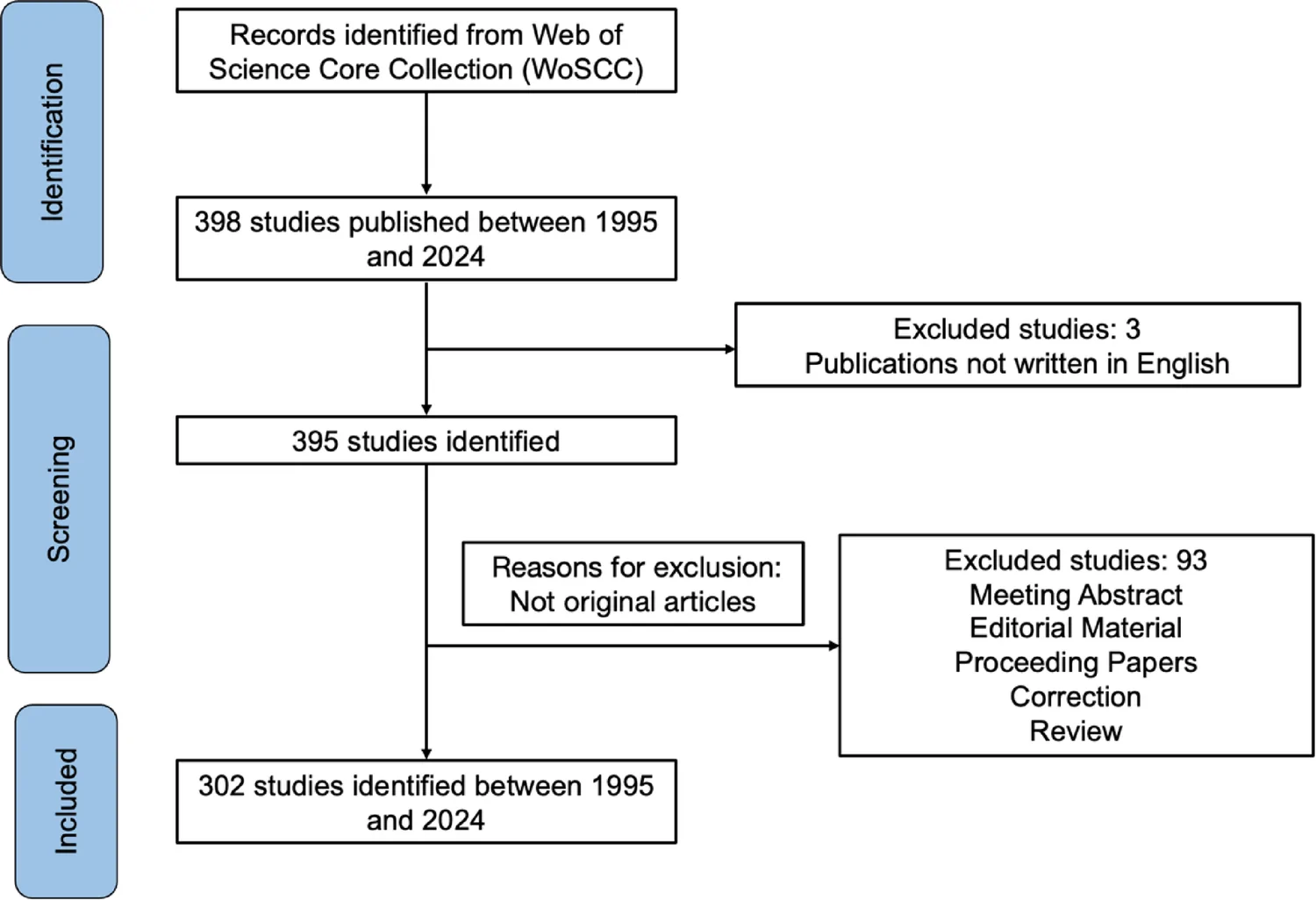
PRISMA Flowchart of screening process concerning relevant publications on CDH

Relevant studies on CDH model research spanning from 1995 to 2024 were retrieved from the Web of Science Core Collection (WoSCC) (Clarivate Analytics, Boston, MA, USA) in July 2025. To ensure the selection of only the most pertinent research outputs, a search strategy focusing on "titles" rather than "topics" was adopted [24, 27]. The following linked search terms were used: “congenital diaphragm hernia” OR “congenital diaphragm defect” OR “fetal diaphragm hernia” OR “pediatric diaphragm hernia” OR “foetal diaphragm hernia” OR “paediatric diaphragm hernia” OR “agenes diaphragm” OR “agenes hemidiaphragm” OR “bochdalek hernia” OR “Morgagni hernia” AND “sheep” OR “nitrofen” OR “lamb” OR “experimental” OR “experiment” OR “animal” OR “model” OR “murine” OR “mice” OR “mouse” OR “rodent” OR “rodents” OR “pig” OR “rat” OR “piglet” OR “rabbit” OR “quail” OR “primate” OR “baboon” OR “Ovine” OR “Dam” [25, 26, 28, 29]. The search was confined to original articles, with publications restricted to the English language. Conference abstract, editorials, corrections, and review articles were excluded from the present analysis. The literature retrieval and screening process strictly followed the PRISMA guideline [30]; the full screening workflow is provided in Fig. 1.

### Distribution and collaboration analysis

Year of publication, citation number, journals, institutions, countries, funding, and authors for each publication were recorded. To examine the evolutionary trends of CDH model research and identify influential publications across different periods, we conducted a temporal segmentation analysis based on decades (1995-2004, 2005-2014, and 2015-2024). This approach aimed to capture research focus and citation patterns over time. Publications affiliated with at least two countries (as determined by the authors institution) were considered as multinational. Funding information for publications was collected from the WoSCC.

### Model type and article topic

Following the screening of abstracts or full texts of the selected articles, the main model type of CDH for each publication was identified. Model types were classified as follows: (1) rat, (2) sheep, (3) rabbit, (4) mouse, (5) porcine model, (6) combined animal model, (7) surgical training model, (8) cell-based in vitro model, and (9) mathematical model (e.g., risk prediction model). To determine the topic of each article, two authors (BS and OA) independently reviewed each paper and identified the main topic of the publication. Any ambiguities arising during the topic identification process were resolved through consensus among all authors. The topic of each publication was classified as follows: (1) developmental biology, (2) surgical technique, (3) physiology (pulmonary hypertension), (4) physiology (oxygenation), (5) physiology (diaphragm development), (6) physiology (cardiac function), (7) gene & protein expression analysis, and (8) others.

### Statistical and bibliometric analysis

Statistical analyses were conducted using GraphPad Prism v.9.0.1 (GraphPad Software, La Jolla, CA, USA). The Spearman correlation coefficient was employed to evaluate relationships between continuous variables. The Kruskal–Wallis test was applied for comparing non-parametric data across different groups, with a *P* value < 0.05 considered statistically significant. Visualization analyses were made with VOSviewer 1.6.20. Keyword co-occurrence analysis (KCA) was performed to examine research topics and the identify connections between items based on their co-occurrence frequency in publications [31]. The dimensional parameters of nodes, together with line thickness, indicate the frequency and strength of their interconnections. The geometric size of nodes corresponds to their respective dimensions, while the nodes themselves represent distinct components [31, 32].

## Results

### Global research productivity

A total of 302 original articles utilizing CDH models, ranging from basic science to surgical models and other experimental setups were identified (Supplementary table). Initial searches of the WoSCC yielded 398 records published between 1995 and 2024. Three non-English publications were excluded, leaving 395 studies for further screening. After excluding 93 records of non-original article types (including review, meeting abstracts, editorial materials, proceeding papers, and corrections), of 302 original articles investigating CDH research models were included for subsequent analysis. These publications originated from 352 institutions in 21 countries. The number of articles published increased significantly over the past three decades, from 85 in 1995-2004 to 127 in the last decade, as verified by Kruskal–Wallis test for inter-decade comparison (*P* < 0.0001). However, both the total number and the mean number of citations per article have declined, from 3,187 and 37 to 980 and 8, respectively. The article entitled “Dual-hit hypothesis explains pulmonary hypoplasia in the nitrofen model of congenital diaphragmatic hernia” published in 2000 in the journal *American Journal of Pathology* by Keijzer et al. was the most cited article from the first decade and overall [33]. This study showed that pulmonary hypoplasia in the toxicologically induced nitrofen model was caused by two separate insults: a direct, primary inhibitory effect of nitrofen on early lung branching morphogenesis and epithelial differentiation, followed by a secondary insult of physical compression after diaphragmatic defect formation. In the second decade (2005-2014), the most cited article was published in 2005 by You et al. in *PNAS.* It identified COUP-TFII deletion in foregut mesenchyme as a cause of Bochdalek-type CDH in mice and a potential contributor to human CDH [34]. “Prenatal microRNA miR-200b Therapy Improves Nitrofen-induced Pulmonary Hypoplasia Associated With Congenital Diaphragmatic Hernia”, published in 2019 by Khoshgoo N et al. in *Annals of Surgery* was the most cited article in the third decade (2015-2024) [35]. This article indicated that prenatal transplacental administration of miR-200b mimics rescued lung hypoplasia and reduced the incidence of CDH in the nitrofen rat model.

### Analysis of journals, institutions, and countries

Articles utilizing CDH models were published in 70 different journals. The *Journal of Pediatric Surgery* published the majority of manuscripts (n = 85, 28%), followed by *Pediatric Surgery International* (n = 61, 20%) and the *American Journal of Physiology Lung Cellular and Molecular Physiology* (*n* = 14, 5%). Most articles (*n* = 158, 52%) originated from pediatric surgery journals, followed by basic science journals (n = 63, 21%) and pediatric journals (*n* = 27, 9%). The impact factor (IF) of most studies (*n* = 219, 73%) was less than three, and only eight (*n* = 8, 3%) articles had an IF of more than ten.

**Fig. 2:**
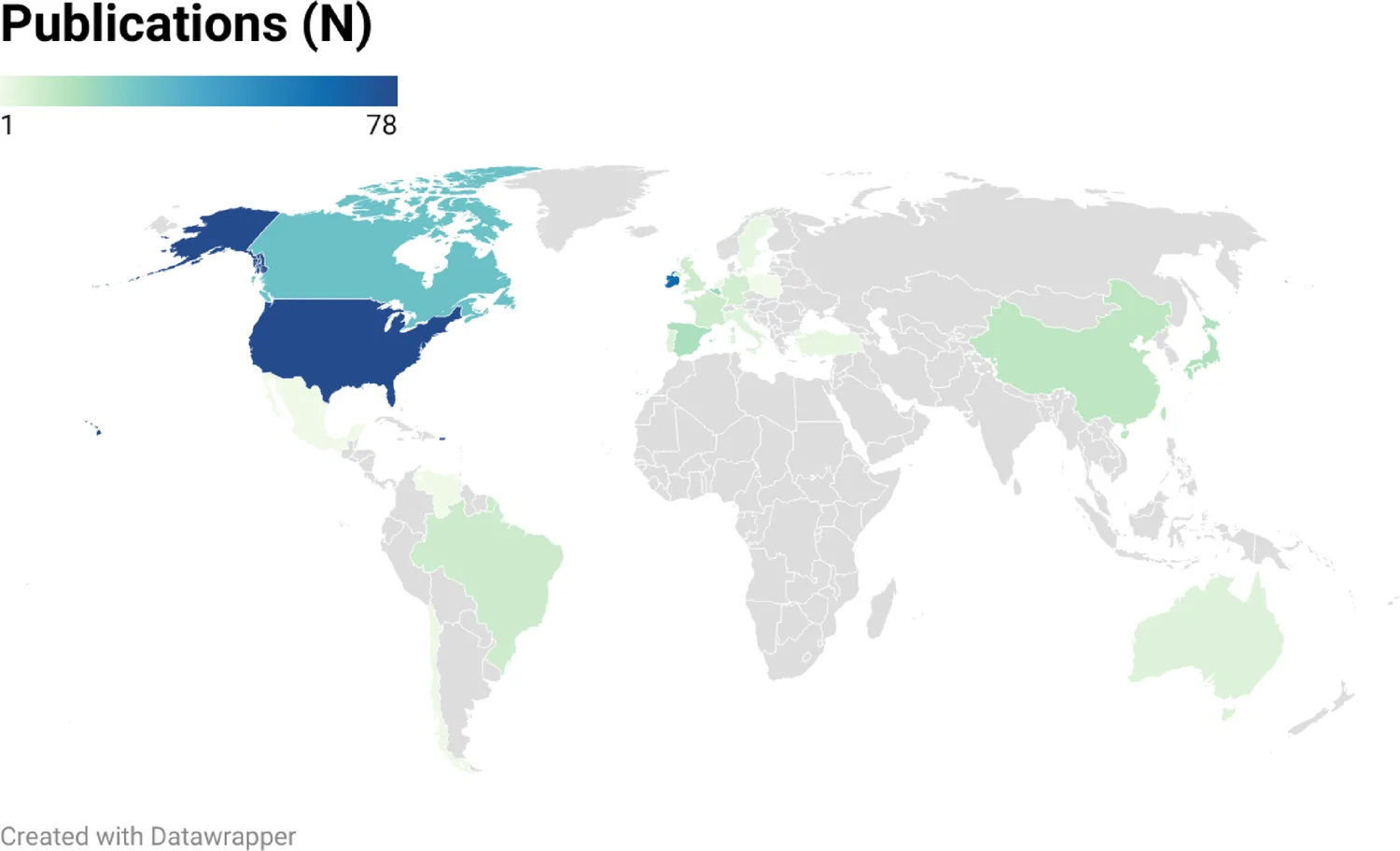
Number of publications on CDH research models per country

Among the institutions and countries contributing to CDH research, the USA published the majority of papers (*n* = 78, 26%), followed by Ireland (*n* = 66, 22%) and Canada (*n* = 30, 10%) (Fig. 2). Most countries were located in North America and Europe. Frequent international collaborations were observed between the USA, Ireland, Germany, England, Belgium, and Canada.

### Analysis of model type and topic

**Fig. 3:**
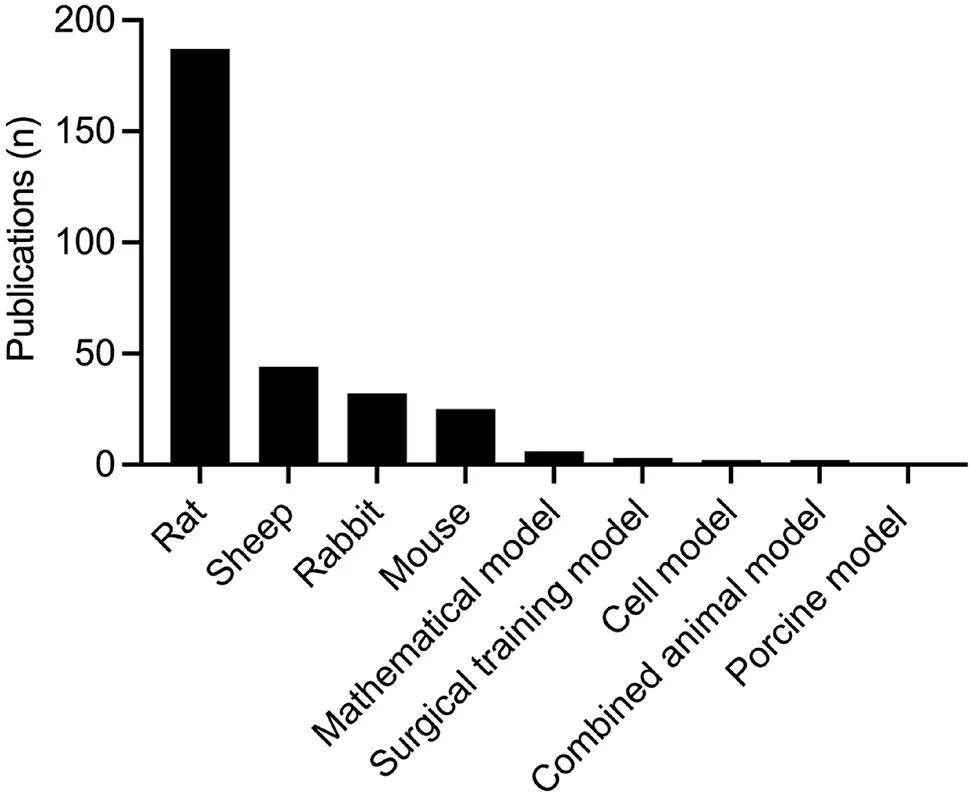
CDH model type distribution over the past three decades (1995 to 2024)

**Fig. 4:**
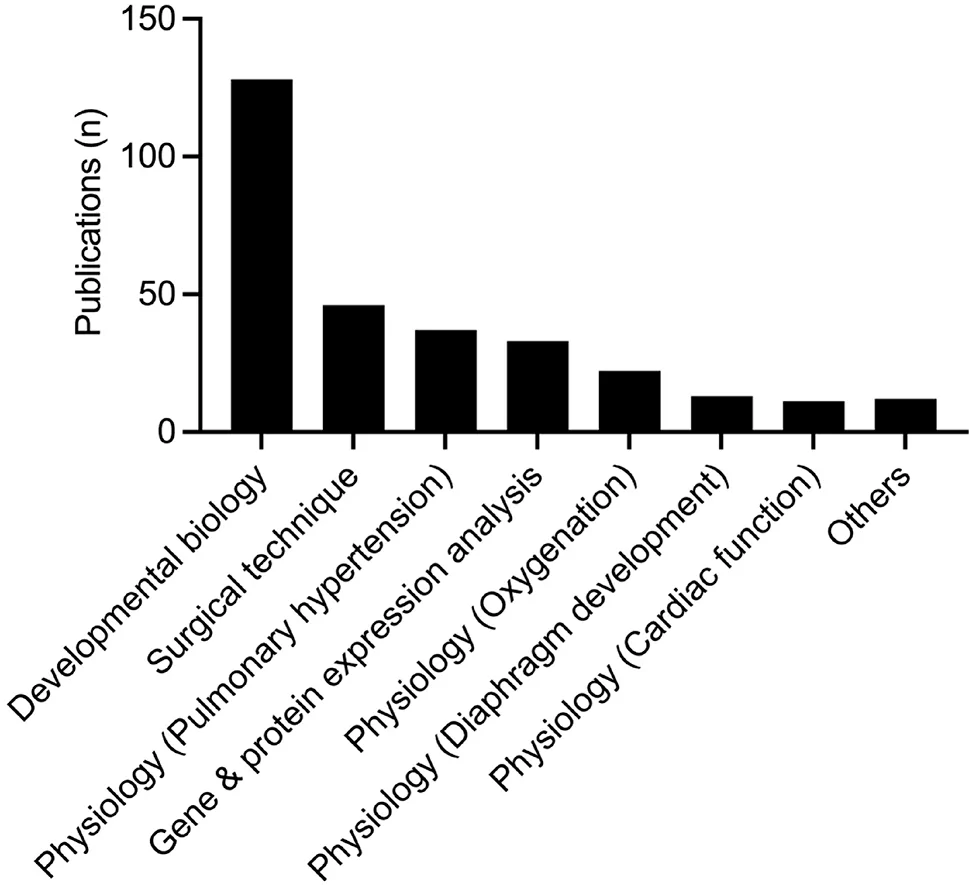
Topics of publications focusing on CDH research models

CDH models were classified in nine different types. The nitrofen rat model (*n* = 187, 62%) was the most commonly used CDH model, followed by the sheep (*n* = 44, 15%) and rabbit model (*n* = 32, 11%) (Fig. 3). Other models used less frequently are outlined in Table 1. The proportion of studies employing the rat model stayed stable over time. Conversely, utilization of the sheep model decreased, while rabbit and mouse models gained popularity (Table 1). Wilcoxon tests were applied to assess time-dependent shifts in the proportional utilization of experimental models. Only the rabbit and mouse model showed significant increase in use over time (*P* < 0.05); no significant temporal changes were found for other model types (*P* > 0.05). Regarding the distribution of article topics, developmental biology occupied the primary position (*n* = 128, 42%), followed by surgical techniques (*n* = 46, 15%) and physiology (with a focus on pulmonary hypertension) (*n* = 37, 12%) (Fig. 4). Within the field of developmental biology, several subtopics were identified. Among them, epithelial cells were most commonly focused on (*n* = 40, 13%), followed by pulmonary hypoplasia (*n* = 26, 9%) and mesenchymal cells (*n* = 17, 6%).

**Table 1:** Number of publications and citations for each CDH research model per decade

Decade	Nitrofen rat model Publications/Citations (*n*)	Sheep model Publications/Citations (*n*)	Rabbit model Publications/Citations (*n*)	Mouse model Publications/Citations (*n*)	Mathematical model Publications/Citations (*n*)	Surgical training model Publications/Citations (*n*)	Combined animal model Publications/Citations (*n*)	Cell model Publications/Citations (*n*)	Porcine model Publications/Citations (*n*)
1995–2004	52 (61.2%)/1921	23 (27.1%)/716	5 (5.9%)/160	4 (4.7%)/351	0 (0%)/0	0 (0%)/0	1 (1.2%)/39	0 (0%)/0	0 (0%)/0
2005–2014	60 (66.7%)/909	9 (10.0%)/121	10 (11.1%)/148	7 (7.8%)/348	1 (1.1%)/26	0 (0%)/0	1 (1.1%)/43	1 (1.1%)/2	1 (1.1%)/18
2015–2024	75 (59.1%)/558	12 (9.4%)/89	17 (13.4%)/124	14 (11.0%)/126	5 (3.9%)/33	3 (2.4%)/26	0 (0%)/0	1 (0.8%)/24	0 (0%)/0

### Analysis of keywords and research trends

**Fig. 5:**
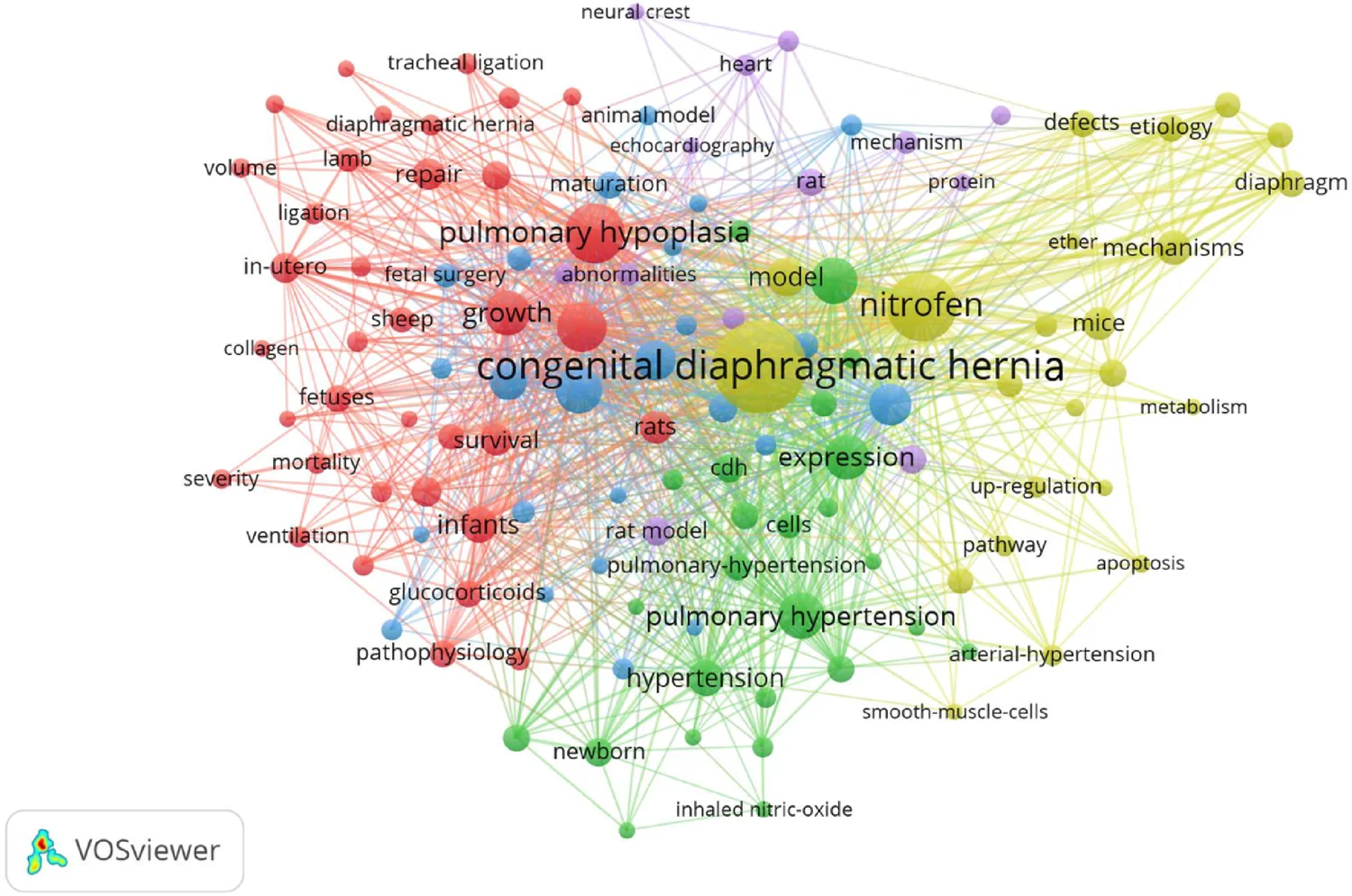
Co-occurrence analysis of keywords relevant to CDH research models; Line thickness and dot size increase respectively with the number of co-occurrences and usage

The VOSviewer was used to identify current research hotspots and emerging trend in the field. Keywords with a minimum occurrence of five were included, resulting in 121 nodes from a total of 1,284 keywords. These were used to generate a co-occurrence network map, as presented in Fig. 5. The top keywords with the highest occurrence were “Congenital diaphragmatic hernia” (*n* = 205), “Nitrofen” (*n* = 105), and “Pulmonary hypoplasia” (n = 79), while "Sildenafil", "Mice", and "Endothelial" were recent research hotspots, which may indicate future areas of interest in this field.

## Discussion

We analyzed global research activity on CDH models over the past three decades using bibliometric methods. A total of 302 articles from 352 institutions across 21 countries were identified, showing steady growth in publication volume and diversity of models. The sustained interest in CDH research highlights its ongoing relevance and emphasizes that it remains a significant challenge in neonatology and pediatric surgery. International and multi-institutional collaboration have expanded. The United States led both in publication output and collaboration networks, consistent with other biomedical fields [36–38]. Research activity was concentrated in North America and Europe, likely reflecting advanced infrastructure, expertise, and funding [39]. In contrast, contributions from developing countries were limited, likely attributable to disparities in resources and governance.

Over the past three decades, CDH model diversity has expanded, initially dominated by rats and sheep, with rabbits and mice gaining popularity in recent years. This shift stems from genetic manipulation advancements and cost constraints favoring rodent over large-animal models [40, 41]. Rodents have been pivotal for elucidating developmental/molecular mechanisms, while sheep, which are valued for anatomical similarity to human fetuses, support interventional studies [28, 29, 42]. However, model diversification has yielded heterogeneous translational outcomes: rodent molecular insights advance pathogenesis understanding, but fail to fully replicate human pathophysiology, while sheep-based surgical innovations inform fetal interventions yet face high costs and do not replicate the double-hit hypothesis[33, 43, 44]. The nitrofen rat model remains widely used for mechanistic pulmonary hypoplasia research but cannot recapitulate all human CDH etiologies [45]. Persistent barriers include species-specific physiological differences, inadequate in vivo integration, and a lack of standardized validation, which impede translation of preclinical findings to improved CDH patient outcomes [40].

Ethical concerns and translational limitations of animal research have stimulated the development of alternative approaches. Recent mathematical models have become indispensable tools in the management of CDH and are primarily used for risk stratification and prognostication [46, 47]. Early models, such as the CDHSG model, served as an initial attempt for predicting mortality risk based on key physiological parameters [46]. However, the field has evolved towards increasingly specific prediction tools [47]. Novel prediction models for the early postnatal period integrate variables available immediately after birth to offer a rapid, individualized estimate of survival probability [48]. Furthermore, they allow for a more nuanced evaluation of how prenatal interventions might alter an infant's predicted postnatal path [49].

Integrating animal models with computational approaches holds promise for advancing CDH research [8]. Animal models capture in vivo physiological dynamics and can be augmented by computational simulations that map omics data, including genetic variants, gene expression profiles, and protein interactions to enable a more comprehensive understanding of CDH pathogenesis. In vitro systems such as patient-derived airway basal stem cell models and organoid models recapitulate lung development and offer physiologically relevant platforms to study CDH at the cellular level [50]. The long-term goal is to use specific genetic data of patients to personalize predictions and therapies, though this remains an emerging area of research [51].

Topic analysis identified developmental biology, surgical techniques, and pulmonary hypertension as the main research foci. Within developmental biology, abnormalities in epithelial and mesenchymal cells were most frequently examined. Disrupted signaling between these compartments leads to impaired lung growth and developmental abnormalities that drive pulmonary hypoplasia and hypertension in CDH, conditions that remain major and unresolved contributors to mortality and long-term morbidity [52, 53].

Research trends include the use of sildenafil for treating pulmonary hypertension, but its clinical benefits, particularly after maternal administration, are still to be determined [54–57]. This therapeutic direction is supported by foundational research validating human findings and a growing recognition of endothelial dysfunction as a core disease mechanism, revealing new potential targets [44, 58].Ultimately, translating these findings will depend on integrating mechanistic research with robust preclinical trials.

## Limitation of the study

This study has several limitations. Restriction to Web of Science and English-language original articles may have led to omissions, and the title-based search strategy, while specific, may have excluded relevant work. In the meantime, bibliometric studies invariably mirror the literature landscape at the time of analysis and are unable to exclude the time-related effects of newly published works and subsequent citations. Nevertheless, the analysis provides an overview of research trends, hotspots, and emerging directions in CDH model research.

## Conclusion

This study mapped research activity and collaboration on CDH models over the past three decades. International cooperation and diversification of models have increased, with rodent and mouse models being particularly relevant for mechanistic studies. Persistent challenges include the limitations of animal models, the difficulty of infant research, and the incomplete understanding of disease etiology. Rigorous studies integrating animal, computational, and human-derived in vitro models will be necessary to advance knowledge and improve translational relevance.

## Supplementary Information

Below is the link to the electronic supplementary material.

Supplementary file1 (DOCX 81 kb)

## Data Availability

The data that supports the findings of this study is available from the author based on reasonable request.

## Abbreviations

CDH: Congenital diaphragmatic hernia
IF: Impact factor
KCA: Keyword co-occurrence analysis
WoSCC: Web of Science Core Collection
