# Supplementary material for: Bibliometric analysis of congenital diaphragmatic hernia research models: evolution and insights from the past three decades
Source: Pediatr Surg Int. 2026 Aug 19;42(1):346. doi: 10.1007/s00383-026-06597-y (PMC13490210; doi:10.1007/s00383-026-06597-y)
Supplement: Supplementary file 1 [file 383_2026_6597_MOESM1_ESM.docx]

**Supplementary table.** All 302 publications related to CDH model.

| **Rank** | **Title** | **Journal** | **Total citations (n)** | **Year** | **Impact Index** | **Impact Factor** | **Country** |
| --- | --- | --- | --- | --- | --- | --- | --- |
| 1 | Dual-hit hypothesis explains pulmonary hypoplasia in the nitrofen model of congenital diaphragmatic hernia | AMERICAN JOURNAL OF PATHOLOGY | 278 | 2000 | 9.0 | 3.6 | Canada |
| 2 | Mouse lacking COUP-TFII as an animal model of Bochdalek-type congenital diaphragmatic hernia | PROCEEDINGS OF THE NATIONAL ACADEMY OF SCIENCES OF THE UNITED STATES OF AMERICA | 135 | 2005 | 14.8 | 9.1 | USA |
| 3 | Antenatal Sildenafil Treatment Attenuates Pulmonary Hypertension in Experimental Congenital Diaphragmatic Hernia | CIRCULATION | 118 | 2011 | 11.9 | 38.6 | Canada |
| 4 | Vitamin A decreases the incidence and severity of nitrofen-induced congenital diaphragmatic hernia in rats | AMERICAN JOURNAL OF PHYSIOLOGY-LUNG CELLULAR AND MOLECULAR PHYSIOLOGY | 115 | 1999 | 22.6 | 3.5 | France |
| 5 | A genetic model for a central (septum transversum) congenital diaphragmatic hernia in mice lacking Slit3 | PROCEEDINGS OF THE NATIONAL ACADEMY OF SCIENCES OF THE UNITED STATES OF AMERICA | 111 | 2003 | 19.8 | 9.1 | USA |
| 6 | Retinal dehydrogenase-2 is inhibited by compounds that induce congenital diaphragmatic hernias in rodents | AMERICAN JOURNAL OF PATHOLOGY | 108 | 2003 | 20.4 | 3.6 | Germany |
| 7 | Mechanisms of action of the congenital diaphragmatic hernia-inducing teratogen nitrofen | AMERICAN JOURNAL OF PHYSIOLOGY-LUNG CELLULAR AND MOLECULAR PHYSIOLOGY | 99 | 2007 | 18.2 | 3.5 | Canada |
| 8 | Congenital diaphragmatic hernia, kidney agenesis and cardiac defects associated with Slit3-deficiency in mice | MECHANISMS OF DEVELOPMENT | 98 | 2003 | 22.4 | 2 | USA |
| 9 | Diaphragm defects occur in a CDH hernia model independently of myogenesis and lung formation | AMERICAN JOURNAL OF PHYSIOLOGY-LUNG CELLULAR AND MOLECULAR PHYSIOLOGY | 94 | 2002 | 24.5 | 3.5 | Canada |
| 10 | Pathogenesis of nitrofen-induced congenital diaphragmatic hernia in fetal rats | JOURNAL OF APPLIED PHYSIOLOGY | 88 | 1997 | 31.8 | 3.3 | Canada |
| 11 | Lung hypoplasia in the nitrofen model of congenital diaphragmatic hernia occurs early in development | AMERICAN JOURNAL OF PHYSIOLOGY-LUNG CELLULAR AND MOLECULAR PHYSIOLOGY | 86 | 2000 | 29.1 | 3.5 | USA |
| 12 | Experimental fetal tracheal ligation and congenital diaphragmatic hernia: a pulmonary vascular morphometric analysis | JOURNAL OF PEDIATRIC SURGERY | 86 | 1995 | 34.9 | 2.5 | USA |
| 13 | Mouse model reveals the role of SOX7 in the development of congenital diaphragmatic hernia associated with recurrent deletions of 8p23.1 | HUMAN MOLECULAR GENETICS | 73 | 2012 | 17.8 | 3.2 | USA |
| 14 | Pulmonary endothelial nitric oxide synthase gene expression is decreased in a rat model of congenital diaphragmatic hernia | AMERICAN JOURNAL OF RESPIRATORY CELL AND MOLECULAR BIOLOGY | 72 | 1995 | 41.7 | 5.3 | USA |
| 15 | Cardiovascular malformations in congenital diaphragmatic hernia: Human and experimental studies | JOURNAL OF PEDIATRIC SURGERY | 71 | 1999 | 36.6 | 2.5 | Spain |
| 16 | Prenatal glucocorticoid therapy reverses pulmonary immaturity in congenital diaphragmatic hernia in fetal sheep | ANNALS OF SURGERY | 67 | 1996 | 43.3 | 6.4 | USA |
| 17 | Early Development of the Primordial Mammalian Diaphragm and Cellular Mechanisms of Nitrofen-Induced Congenital Diaphragmatic Hernia | BIRTH DEFECTS RESEARCH PART A-CLINICAL AND MOLECULAR TERATOLOGY | 64 | 2010 | 23.4 | 1.6 | Canada |
| 18 | Perfluorocarbon-associated gas exchange improves pulmonary mechanics, oxygenation, ventilation, and allows nitric oxide delivery in the hypoplastic lung congenital diaphragmatic hernia lamb model | CRITICAL CARE MEDICINE | 62 | 1995 | 48.4 | 6 | USA |
| 19 | Prenatal hormonal therapy improves pulmonary compliance in the nitrofen-induced CDH rat model | JOURNAL OF PEDIATRIC SURGERY | 58 | 1995 | 51.7 | 2.5 | USA |
| 20 | Pulmonary vascular morphology in a fetal rabbit model for congenital diaphragmatic hernia | JOURNAL OF PEDIATRIC SURGERY | 57 | 2004 | 36.8 | 2.5 | Belgium |
| 21 | Restoring effects of vitamin A on surfactant synthesis in nitrofen-induced congenital diaphragmatic hernia in rats | AMERICAN JOURNAL OF RESPIRATORY AND CRITICAL CARE MEDICINE | 55 | 2001 | 43.6 | 19.4 | France |
| 22 | Pathophysiology of congenital diaphragmatic hernia. XI: Anatomic and biochemical characterization of the heart in the fetal lamb CDH model | JOURNAL OF PEDIATRIC SURGERY | 54 | 1995 | 55.6 | 2.5 | USA |
| 23 | Effect of tracheal occlusion on peripheric pulmonary vessel muscularization in a fetal rabbit model for congenital diaphragmatic hernia | AMERICAN JOURNAL OF OBSTETRICS AND GYNECOLOGY | 52 | 2004 | 40.4 | 8.4 | Belgium |
| 24 | Candidate genes for congenital diaphragmatic hernia from animal models:: sequencing of FOG2 and PDGFRα reveals rare variants in diaphragmatic hernia patients | EUROPEAN JOURNAL OF HUMAN GENETICS | 51 | 2007 | 35.3 | 4.6 | USA |
| 25 | Prenatal microRNA miR-200b Therapy Improves Nitrofen-induced Pulmonary Hypoplasia Associated With Congenital Diaphragmatic Hernia | ANNALS OF SURGERY | 50 | 2019 | 12.0 | 6.4 | Canada |
| 26 | VEGF expression is downregulated in nitrofen-induced congenital diaphragmatic hernia | JOURNAL OF PEDIATRIC SURGERY | 48 | 2004 | 43.8 | 2.5 | USA |
| 27 | Recent advances in understanding the pathogenesis of nitrofen-induced congenital diaphragmatic hernia | PEDIATRIC PULMONOLOGY | 48 | 2000 | 52.1 | 2.3 | Canada |
| 28 | Effect of prenatal glucocorticoids on pulmonary vascular muscularisation in nitrofen-induced congenital diaphragmatic hernia | JOURNAL OF PEDIATRIC SURGERY | 47 | 1998 | 57.4 | 2.5 | UK |
| 29 | Heart hypoplasia in experimental congenital diaphragmatic hernia | JOURNAL OF PEDIATRIC SURGERY | 46 | 1999 | 56.5 | 2.5 | Spain |
| 30 | Prenatal treatment with retinoic acid promotes pulmonary alveologenesis in the nitrofen model of congenital diaphragmatic hernia | JOURNAL OF PEDIATRIC SURGERY | 45 | 2008 | 37.8 | 2.5 | Ireland |
| 31 | Fetal tracheal occlusion in the rat model of nitrofen-induced congenital diaphragmatic hernia: Tracheal occlusion reverses the arterial structural abnormality | JOURNAL OF PEDIATRIC SURGERY | 44 | 2001 | 54.5 | 2.5 | USA |
| 32 | Decreased lung fibroblast growth factor 18 and elastin in human congenital diaphragmatic hernia and animal models | AMERICAN JOURNAL OF RESPIRATORY AND CRITICAL CARE MEDICINE | 43 | 2007 | 41.9 | 19.4 | France |
| 33 | Effect of antenatal tracheal occlusion on lung development in the sheep model of congenital diaphragmatic hernia: A morphometric analysis of pulmonary structure and maturity | PEDIATRIC PULMONOLOGY | 43 | 1998 | 62.8 | 2.3 | Australia |
| 34 | Pathophysiology of congenital diaphragmatic hernia. VIII: Inhaled nitric oxide requires exogenous surfactant therapy in the lamb model of congenital diaphragmatic hernia | JOURNAL OF PEDIATRIC SURGERY | 41 | 1995 | 73.2 | 2.5 | USA |
| 35 | Structure of the primordial diaphragm and defects associated with nitrofen-induced CDH | JOURNAL OF APPLIED PHYSIOLOGY | 41 | 2000 | 61.0 | 3.3 | Canada |
| 36 | Conditional deletion of WT1 in the septum transversum mesenchyme causes congenital diaphragmatic hernia in mice | ELIFE | 40 | 2016 | 22.5 | NA | Spain |
| 37 | Ghrelin expression in human and rat fetal lungs and the effect of ghrelin administration in nitrofen-induced congenital diaphragmatic hernia | PEDIATRIC RESEARCH | 40 | 2006 | 47.5 | 3.1 | Portugal |
| 38 | Surfactant protein A is decreased in a rat model of congenital diaphragmatic hernia | AMERICAN JOURNAL OF RESPIRATORY AND CRITICAL CARE MEDICINE | 40 | 1998 | 67.5 | 19.4 | USA |
| 39 | Lung tissue mechanics predict lung hypoplasia in a rabbit model for congenital diaphragmatic hernia | PEDIATRIC PULMONOLOGY | 39 | 2007 | 46.2 | 2.3 | Germany |
| 40 | Animal models in congenital diaphragmatic hernia | CLINICS IN PERINATOLOGY | 39 | 1996 | 74.4 | 2.4 | UK |
| 41 | Balloon tracheal occlusion for congenital diaphragmatic hernia: Experimental studies | JOURNAL OF PEDIATRIC SURGERY | 38 | 2000 | 65.8 | 2.5 | USA |
| 42 | Cardiovascular malformations in experimental congenital diaphragmatic hernia | JOURNAL OF PEDIATRIC SURGERY | 38 | 1999 | 68.4 | 2.5 | UK |
| 43 | Surfactant decreases pulmonary vascular resistance and increases pulmonary blood flow in the fetal lamb model of congenital diaphragmatic hernia | JOURNAL OF PEDIATRIC SURGERY | 38 | 1996 | 76.3 | 2.5 | USA |
| 44 | Lung growth induced by prenatal tracheal occlusion and its modifying factors: A study in the rat model of congenital diaphragmatic hernia | JOURNAL OF PEDIATRIC SURGERY | 37 | 2001 | 64.9 | 2.5 | USA |
| 45 | Fetal tracheal occlusion in the rat model of nitrofen-induced congenital diaphragmatic hernia | JOURNAL OF APPLIED PHYSIOLOGY | 37 | 1999 | 70.3 | 3.3 | USA |
| 46 | Deficiency of FRAS1-related extracellular matrix 1 (FREM1) causes congenital diaphragmatic hernia in humans and mice | HUMAN MOLECULAR GENETICS | 36 | 2013 | 33.3 | 3.2 | USA |
| 47 | Decreased pulmonary nitric oxide synthase activity in the rat model of congenital diaphragmatic hernia | JOURNAL OF PEDIATRIC SURGERY | 36 | 1996 | 80.6 | 2.5 | USA |
| 48 | Prenatal treatment with retinoic acid accelerates type 1 alveolar cell proliferation of the hypoplastic lung in the nitrofen model of congenital diaphragmatic hernia | JOURNAL OF PEDIATRIC SURGERY | 35 | 2008 | 48.6 | 2.5 | Ireland |
| 49 | Neural crest-derived defects in experimental congenital diaphragmatic hernia | PEDIATRIC SURGERY INTERNATIONAL | 35 | 2001 | 68.6 | 1.6 | Spain |
| 50 | Administration of antenatal glucocorticoids prevents pulmonary artery structural changes in nitrofen-induced congenital diaphragmatic hernia in rats | JOURNAL OF PEDIATRIC SURGERY | 35 | 1998 | 77.1 | 2.5 | Ireland |
| 51 | Myeloperoxidase activity as a lung injury marker in the lamb model of congenital diaphragmatic hernia | JOURNAL OF PEDIATRIC SURGERY | 33 | 2001 | 72.7 | 2.5 | USA |
| 52 | Increased vascular endothelial growth factor peptide and gene expression in hypoplastic lung in nitrofen induced congenital diaphragmatic hernia in rats | PEDIATRIC SURGERY INTERNATIONAL | 32 | 2002 | 71.9 | 1.6 | Ireland |
| 53 | Prenatal glucocorticoids improve pulmonary morphometrics in fetal sheep with congenital diaphragmatic hernia | JOURNAL OF PEDIATRIC SURGERY | 32 | 1997 | 87.5 | 2.5 | USA |
| 54 | Embryonic cell death patterns associated with nitrofen-induced congenital diaphragmatic hernia | JOURNAL OF PEDIATRIC SURGERY | 32 | 1995 | 93.8 | 2.5 | USA |
| 55 | Assessment of the nitrofen model of congenital diaphragmatic hernia and of the dysregulated factors involved in pulmonary hypoplasia | PEDIATRIC SURGERY INTERNATIONAL | 31 | 2019 | 19.4 | 1.6 | Canada |
| 56 | Skeletal malformations associated with congenital diaphragmatic hernia: Experimental and human studies | JOURNAL OF PEDIATRIC SURGERY | 31 | 1999 | 83.9 | 2.5 | Spain |
| 57 | Pulmonary artery endothelial cell dysfunction and decreased populations of highly proliferative endothelial cells in experimental congenital diaphragmatic hernia | AMERICAN JOURNAL OF PHYSIOLOGY-LUNG CELLULAR AND MOLECULAR PHYSIOLOGY | 30 | 2013 | 40.0 | 3.5 | USA |
| 58 | Kif7 is required for the patterning and differentiation of the diaphragm in a model of syndromic congenital diaphragmatic hernia | PROCEEDINGS OF THE NATIONAL ACADEMY OF SCIENCES OF THE UNITED STATES OF AMERICA | 30 | 2013 | 40.0 | 9.1 | USA |
| 59 | Pulmonary surfactant protein A, B, and C mRNA and protein expression in the nitrofen-induced congenital diaphragmatic hernia rat model | PEDIATRIC RESEARCH | 30 | 2003 | 73.3 | 3.1 | Netherlands |
| 60 | Effect of antenatal glucocorticoid administration on insulin-like growth factor I and II levels in hypoplastic lung in nitrofen-induced congenital diaphragmatic hernia in rats | PEDIATRIC SURGERY INTERNATIONAL | 30 | 1999 | 86.7 | 1.6 | Ireland |
| 61 | The use of human amniotic fluid stem cells as an adjunct to promote pulmonary development in a rabbit model for congenital diaphragmatic hernia | PRENATAL DIAGNOSIS | 28 | 2015 | 35.7 | 2.7 | Belgium |
| 62 | Prenatal retinoic acid improves lung vascularization and VEGF expression in CDH rat | AMERICAN JOURNAL OF OBSTETRICS AND GYNECOLOGY | 28 | 2012 | 46.4 | 8.4 | USA |
| 63 | Improved pulmonary function in the nitrofen model of congenital diaphragmatic hernia following prenatal maternal dexamethasone and/or sildenafil | PEDIATRIC RESEARCH | 27 | 2016 | 33.3 | 3.1 | USA |
| 64 | ETA-receptor blockade and ETB-receptor stimulation in experimental congenital diaphragmatic hernia | AMERICAN JOURNAL OF PHYSIOLOGY-LUNG CELLULAR AND MOLECULAR PHYSIOLOGY | 27 | 2000 | 92.6 | 3.5 | France |
| 65 | Multivariate model for predicting recurrence in congenital diaphragmatic hernia | JOURNAL OF PEDIATRIC SURGERY | 26 | 2009 | 61.5 | 2.5 | USA |
| 66 | Administration of antenatal glucocorticoids upregulates peptide growth factor gene expression in nitrofen-induced congenital diaphragmatic hernia in rats | JOURNAL OF PEDIATRIC SURGERY | 26 | 2000 | 96.2 | 2.5 | Ireland |
| 67 | Surfactant rescue in the fetal lamb model of congenital diaphragmatic hernia | JOURNAL OF PEDIATRIC SURGERY | 26 | 1996 | 111.5 | 2.5 | USA |
| 68 | Treatment of rat congenital diaphragmatic hernia with sildenafil and NS-304, selexipag's active compound, at the pseudoglandular stage improves lung vasculature | AMERICAN JOURNAL OF PHYSIOLOGY-LUNG CELLULAR AND MOLECULAR PHYSIOLOGY | 25 | 2018 | 28.0 | 3.5 | Netherlands |
| 69 | Antenatal imatinib treatment reduces pulmonary vascular remodeling in a rat model of congenital diaphragmatic hernia | AMERICAN JOURNAL OF PHYSIOLOGY-LUNG CELLULAR AND MOLECULAR PHYSIOLOGY | 25 | 2012 | 52.0 | 3.5 | Sweden |
| 70 | Impaired alveolar epithelial cell differentiation in the hypoplastic lung in nitrofen-induced congenital diaphragmatic hernia | PEDIATRIC SURGERY INTERNATIONAL | 25 | 2007 | 72.0 | 1.6 | Ireland |
| 71 | Human induced pluripotent stem cell-derived lung organoids in an ex vivo model of the congenital diaphragmatic hernia fetal lung | STEM CELLS TRANSLATIONAL MEDICINE | 24 | 2021 | 16.7 | 4.9 | USA |
| 72 | Prevention of pulmonary hypoplasia and pulmonary vascular remodeling by antenatal simvastatin treatment in nitrofen-induced congenital diaphragmatic hernia | AMERICAN JOURNAL OF PHYSIOLOGY-LUNG CELLULAR AND MOLECULAR PHYSIOLOGY | 24 | 2015 | 41.7 | 3.5 | Belgium |
| 73 | The relationship of diaphragmatic defect, liver growth, and lung hypoplasia in nitrofen-induced congenital diaphragmatic hernia in the rat | PEDIATRIC SURGERY INTERNATIONAL | 24 | 2004 | 87.5 | 1.6 | Germany |
| 74 | Population-Based Validation of a Clinical Prediction Model for Congenital Diaphragmatic Hernias | JOURNAL OF PEDIATRICS | 23 | 2018 | 30.4 | 3.5 | USA |
| 75 | Abnormal platelet-derived growth factor signaling accounting for lung hypoplasia in experimental congenital diaphragmatic hernia | JOURNAL OF PEDIATRIC SURGERY | 22 | 2010 | 68.2 | 2.5 | Ireland |
| 76 | The tracheobronchial tree is abnormal in experimental congenital diaphragmatic hernia | PEDIATRIC SURGERY INTERNATIONAL | 22 | 1999 | 118.2 | 1.6 | Spain |
| 77 | Effect of dexamethasone on endothelial nitric oxide synthase in experimental congenital diaphragmatic hernia | ARCHIVES OF DISEASE IN CHILDHOOD-FETAL AND NEONATAL EDITION | 22 | 1998 | 122.7 | 3.6 | UK |
| 78 | INTESTINAL ROTATION IN EXPERIMENTAL CONGENITAL DIAPHRAGMATIC-HERNIA | JOURNAL OF PEDIATRIC SURGERY | 22 | 1995 | 136.4 | 2.5 | Spain |
| 79 | Fetal heart development in the nitrofen-induced CDH rat model: The role of mechanical and nonmechanical factors | JOURNAL OF PEDIATRIC SURGERY | 22 | 2003 | 100.0 | 2.5 | Portugal |
| 80 | Antenatal maternally-administered phosphodiesterase type 5 inhibitors normalize eNOS expression in the fetal lamb model of congenital diaphragmatic hernia | JOURNAL OF PEDIATRIC SURGERY | 21 | 2014 | 52.4 | 2.5 | USA |
| 81 | Effects of maternal retinoic acid administration in a congenital diaphragmatic hernia rabbit model | PEDIATRIC PULMONOLOGY | 21 | 2008 | 81.0 | 2.3 | France |
| 82 | Upregulation of keratinocyte growth factor in the tracheal ligation lamb model of congenital diaphragmatic hernia | JOURNAL OF PEDIATRIC SURGERY | 21 | 2001 | 114.3 | 2.5 | USA |
| 83 | The effects of prenatal intraamniotic surfactant or dexamethasone administration on lung development are comparable to changes induced by tracheal ligation in an animal model of congenital diaphragmatic hernia | JOURNAL OF PEDIATRIC SURGERY | 21 | 1998 | 128.6 | 2.5 | Brazil |
| 84 | Neonatal cardiopulmonary transition in an ovine model of congenital diaphragmatic hernia | ARCHIVES OF DISEASE IN CHILDHOOD-FETAL AND NEONATAL EDITION | 20 | 2019 | 30.0 | 3.6 | Australia |
| 85 | Decreased expression of miR-33 in fetal lungs of nitrofen-induced congenital diaphragmatic hernia rat model | JOURNAL OF PEDIATRIC SURGERY | 20 | 2016 | 45.0 | 2.5 | China |
| 86 | Therapeutic potential of mesenchymal stem cell transplantation in a nitrofen-induced congenital diaphragmatic hernia rat model | PEDIATRIC SURGERY INTERNATIONAL | 20 | 2014 | 55.0 | 1.6 | Japan |
| 87 | VEGF receptor expression decreases during lung development in congenital diaphragmatic hernia induced by nitrofen | BRAZILIAN JOURNAL OF MEDICAL AND BIOLOGICAL RESEARCH | 20 | 2014 | 55.0 | 1.5 | Brazil |
| 88 | Morphometric analysis of preterm fetal pulmonary development in the sheep model of congenital diaphragmatic hernia | PEDIATRIC AND DEVELOPMENTAL PATHOLOGY | 20 | 2000 | 125.0 | 1.3 | Australia |
| 89 | Morphometric analysis of pulmonary development in the sheep following creation of fetal diaphragmatic hernia | PEDIATRIC PATHOLOGY & LABORATORY MEDICINE | 20 | 1997 | 140.0 | NA | Australia |
| 90 | Pathophysiology of congenital diaphragmatic hernia. XVI: Elevated pulmonary collagen in the lamb model of congenital diaphragmatic hernia | JOURNAL OF PEDIATRIC SURGERY | 20 | 1995 | 150.0 | 2.5 | USA |
| 91 | Alveolarization Genes Modulated by Fetal Tracheal Occlusion in the Rabbit Model for Congenital Diaphragmatic Hernia: A Randomized Study | PLOS ONE | 19 | 2013 | 63.2 | 2.6 | Belgium |
| 92 | Gene expression analysis in hypoplastic lungs in the nitrofen model of congenital diaphragmatic hernia | JOURNAL OF PEDIATRIC SURGERY | 19 | 2010 | 78.9 | 2.5 | Sweden |
| 93 | Nitrovasoditator responses in pulmonary arteriotes from rats with nitrofen-induced congenital diaphragmatic hernia | JOURNAL OF PEDIATRIC SURGERY | 19 | 2005 | 105.3 | 2.5 | USA |
| 94 | Alveolar vascularization of the lung in a lamb model of congenital diaphragmatic hernia | AMERICAN JOURNAL OF RESPIRATORY AND CRITICAL CARE MEDICINE | 19 | 1998 | 142.1 | 19.4 | USA |
| 95 | An Endoscopic Surgical Skill Validation System for Pediatric Surgeons Using a Model of Congenital Diaphragmatic Hernia Repair | JOURNAL OF LAPAROENDOSCOPIC & ADVANCED SURGICAL TECHNIQUES | 18 | 2015 | 55.6 | 1.1 | Japan |
| 96 | Sildenafil Stimulates and Dexamethasone Inhibits Pulmonary Vascular Development in Congenital Diaphragmatic Hernia Rat Lungs | NEONATOLOGY | 18 | 2014 | 61.1 | 3 | Chile |
| 97 | Absorbable Versus Nonabsorbable Mesh Repair of Congenital Diaphragmatic Hernias in a Growing Animal Model | JOURNAL OF LAPAROENDOSCOPIC & ADVANCED SURGICAL TECHNIQUES | 18 | 2011 | 77.8 | 1.1 | Mexico |
| 98 | Airway smooth muscle changes in the nitrofen-induced congenital diaphragmatic hernia rat model | PEDIATRIC RESEARCH | 18 | 2003 | 122.2 | 3.1 | Canada |
| 99 | Expression of surfactant proteins and thyroid transcription factor 1 in an ovine model of congenital diaphragmatic hernia | JOURNAL OF PEDIATRIC SURGERY | 18 | 2002 | 127.8 | 2.5 | France |
| 100 | In vitro effects of growth factors on lung hypoplasia in a model of congenital diaphragmatic hernia | JOURNAL OF PEDIATRIC SURGERY | 18 | 2000 | 138.9 | 2.5 | UK |
| 101 | Contractile properties of intralobar pulmonary arteries and veins in the fetal lamb model of congenital diaphragmatic hernia | JOURNAL OF PEDIATRIC SURGERY | 18 | 1998 | 150.0 | 2.5 | USA |
| 102 | Treatment with Amniotic Fluid Stem Cell Extracellular Vesicles Promotes Fetal Lung Branching and Cell Differentiation at Canalicular and Saccular Stages in Experimental Pulmonary Hypoplasia Secondary to Congenital Diaphragmatic Hernia | STEM CELLS TRANSLATIONAL MEDICINE | 17 | 2022 | 17.6 | 4.9 | Canada |
| 103 | Proteomic profiling of tracheal fluid in an ovine model of congenital diaphragmatic hernia and fetal tracheal occlusion | AMERICAN JOURNAL OF PHYSIOLOGY-LUNG CELLULAR AND MOLECULAR PHYSIOLOGY | 17 | 2018 | 41.2 | 3.5 | USA |
| 104 | Altered pulmonary artery endothelial-smooth muscle cell interactions in experimental congenital diaphragmatic hernia | PEDIATRIC RESEARCH | 17 | 2015 | 58.8 | 3.1 | USA |
| 105 | Timing and expression of the angiopoietin-1-Tie-2 pathway in murine lung development and congenital diaphragmatic hernia | DISEASE MODELS & MECHANISMS | 17 | 2013 | 70.6 | 3.3 | USA |
| 106 | Lung Tissue Blood Perfusion Changes Induced by in utero Tracheal Occlusion in a Rabbit Model of Congenital Diaphragmatic Hernia | FETAL DIAGNOSIS AND THERAPY | 17 | 2009 | 94.1 | 1.6 | Spain |
| 107 | Antenatal glucocorticoid therapy suppresses angiotensin-converting enzyme activity in rats with nitrofen-induced congenital diaphragmatic hernia | JOURNAL OF PEDIATRIC SURGERY | 17 | 1998 | 158.8 | 2.5 | UK |
| 108 | Kif7 expression is decreased in the diaphragmatic and pulmonary mesenchyme of nitrofen-induced congenital diaphragmatic hernia | JOURNAL OF PEDIATRIC SURGERY | 16 | 2015 | 62.5 | 2.5 | Ireland |
| 109 | Congenital diaphragmatic hernia prevents absorption of distal air space fluid in late-gestation rat fetuses | AMERICAN JOURNAL OF PHYSIOLOGY-LUNG CELLULAR AND MOLECULAR PHYSIOLOGY | 16 | 2006 | 118.8 | 3.5 | USA |
| 110 | The vagus and recurrent laryngeal nerves in experimental congenital diaphragmatic hernia | PEDIATRIC SURGERY INTERNATIONAL | 16 | 2004 | 131.3 | 1.6 | Spain |
| 111 | A comparison of collagen and PTFE patch repair in a rabbit model of congenital diaphragmatic hernia | JOURNAL OF INVESTIGATIVE SURGERY | 16 | 2000 | 156.3 | 3.5 | USA |
| 112 | The effects of tracheal occlusion on Wnt signaling in a rabbit model of congenital diaphragmatic hernia | JOURNAL OF PEDIATRIC SURGERY | 15 | 2019 | 40.0 | 2.5 | Canada |
| 113 | The proportion of alveolar type 1 cells decreases in murine hypoplastic congenital diaphragmatic hernia lungs | PLOS ONE | 15 | 2019 | 40.0 | 2.6 | Belgium |
| 114 | Prenatal treatment with rosiglitazone attenuates vascular remodeling and pulmonary monocyte influx in experimental congenital diaphragmatic hernia | PLOS ONE | 15 | 2018 | 46.7 | 2.6 | Germany |
| 115 | The transcriptome of nitrofen-induced pulmonary hypoplasia in the rat model of congenital diaphragmatic hernia | PEDIATRIC RESEARCH | 15 | 2016 | 60.0 | 3.1 | Canada |
| 116 | Disruption of the Bone Morphogenetic Protein Receptor 2 Pathway in Nitrofen-Induced Congenital Diaphragmatic Hernia | BIRTH DEFECTS RESEARCH PART B-DEVELOPMENTAL AND REPRODUCTIVE TOXICOLOGY | 15 | 2013 | 80.0 | 1.6 | Germany |
| 117 | An intra-amniotic injection of mesenchymal stem cells promotes lung maturity in a rat congenital diaphragmatic hernia model | PEDIATRIC SURGERY INTERNATIONAL | 14 | 2019 | 42.9 | 1.6 | Japan |
| 118 | Echocardiography allows for analysis of pulmonary arterial flow in mice with congenital diaphragmatic hernia | JOURNAL OF SURGICAL RESEARCH | 14 | 2018 | 50.0 | 1.7 | USA |
| 119 | Increased TGF-β: a drawback of tracheal occlusion in human and experimental congenital diaphragmatic hernia? | AMERICAN JOURNAL OF PHYSIOLOGY-LUNG CELLULAR AND MOLECULAR PHYSIOLOGY | 14 | 2016 | 64.3 | 3.5 | Belgium |
| 120 | Decreased apelin and apelin-receptor expression in the pulmonary vasculature of nitrofen-induced congenital diaphragmatic hernia | PEDIATRIC SURGERY INTERNATIONAL | 14 | 2014 | 78.6 | 1.6 | Ireland |
| 121 | Downregulated bone morphogenetic protein signaling in nitrofen-induced congenital diaphragmatic hernia | PEDIATRIC SURGERY INTERNATIONAL | 14 | 2013 | 85.7 | 1.6 | Belgium |
| 122 | Reduced expression of aquaporin 5 water channel in nitrofen-induced hypoplastic lung with congenital diaphragmatic hernia rat model | JOURNAL OF PEDIATRIC SURGERY | 14 | 2007 | 128.6 | 2.5 | Ireland |
| 123 | The effects of prenatal intraamniotic surfactant or dexamethasone administration on lung development are comparable to changes induced by tracheal ligation in an animal model of congenital diaphragmatic hernia: Studies of lung glycogen content, elastic fiber density, and collagen content | JOURNAL OF PEDIATRIC SURGERY | 14 | 1998 | 192.9 | 2.5 | Brazil |
| 124 | A Tracheal Aspirate-derived Airway Basal Cell Model Reveals a Proinflammatory Epithelial Defect in Congenital Diaphragmatic Hernia | AMERICAN JOURNAL OF RESPIRATORY AND CRITICAL CARE MEDICINE | 13 | 2023 | 15.4 | 19.4 | USA |
| 125 | Complementary Effect of Maternal Sildenafil and Fetal Tracheal Occlusion Improves Lung Development in the Rabbit Model of Congenital Diaphragmatic Hernia | ANNALS OF SURGERY | 13 | 2022 | 23.1 | 6.4 | Belgium |
| 126 | ROBO2 signaling in lung development regulates SOX2/SOX9 balance, branching morphogenesis and is dysregulated in nitrofen-induced congenital diaphragmatic hernia | RESPIRATORY RESEARCH | 13 | 2020 | 38.5 | 5 | Portugal |
| 127 | Perturbations in Endothelial Dysfunction-Associated Pathways in the Nitrofen-Induced Congenital Diaphragmatic Hernia Model | JOURNAL OF VASCULAR RESEARCH | 13 | 2018 | 53.8 | 2.3 | USA |
| 128 | Lysyl Oxidase Expression Is Decreased in the Developing Diaphragm and Lungs of Nitrofen-Induced Congenital Diaphragmatic Hernia | EUROPEAN JOURNAL OF PEDIATRIC SURGERY | 13 | 2015 | 76.9 | 1.4 | Ireland |
| 129 | Late administration of antenatal vitamin A promotes pulmonary structural maturation and improves ventilation in the lamb model of congenital diaphragmatic hernia | PEDIATRIC SURGERY INTERNATIONAL | 13 | 2011 | 107.7 | 1.6 | USA |
| 130 | Antenatal steroid and tracheal occlusion restore vascular endothelial growth factor receptors in congenital diaphragmatic hernia rat model | AMERICAN JOURNAL OF OBSTETRICS AND GYNECOLOGY | 13 | 2010 | 115.4 | 8.4 | Brazil |
| 131 | Effect of prenatal tetrandrine administration on transforming growth factor-β1 level in the lung of nitrofen-induced congenital diaphragmatic hernia rat model | JOURNAL OF PEDIATRIC SURGERY | 13 | 2009 | 123.1 | 2.5 | China |
| 132 | Decreased expression of voltage-gated K+ channels in pulmonary artery smooth muscles cells in nitrofen-induced congenital diaphragmatic hernia in rats | PEDIATRIC SURGERY INTERNATIONAL | 13 | 2004 | 161.5 | 1.6 | Ireland |
| 133 | Nitrofen-induced congenital Diaphragmatic hernia in rat embryo: What model? | JOURNAL OF PEDIATRIC SURGERY | 13 | 2004 | 161.5 | 2.5 | Poland |
| 134 | Oxygen-induced vasodilation is blunted in pulmonary arterioles from fetal rats with nitrofen-induced congenital diaphragmatic hernia | JOURNAL OF PEDIATRIC SURGERY | 13 | 2001 | 184.6 | 2.5 | USA |
| 135 | Defective mesothelium and limited physical space are drivers of dysregulated lung development in a genetic model of congenital diaphragmatic hernia | DEVELOPMENT | 12 | 2021 | 33.3 | 3.6 | USA |
| 136 | Extracellular Vesicles Attenuate Nitrofen-Mediated Human Pulmonary Artery Endothelial Dysfunction: Implications for Congenital Diaphragmatic Hernia | STEM CELLS AND DEVELOPMENT | 12 | 2020 | 41.7 | 2 | USA |
| 137 | Increased expression of activated pSTAT3 and PIM-1 in the pulmonary vasculature of experimental congenital diaphragmatic hernia | JOURNAL OF PEDIATRIC SURGERY | 12 | 2015 | 83.3 | 2.5 | Ireland |
| 138 | Prenatal retinoic acid increases lipofibroblast expression in hypoplastic rat lungs with experimental congenital diaphragmatic hernia | JOURNAL OF PEDIATRIC SURGERY | 12 | 2014 | 91.7 | 2.5 | Ireland |
| 139 | Signaling molecules in the fetal rabbit model for congenital diaphragmatic hernia | PEDIATRIC PULMONOLOGY | 12 | 2012 | 108.3 | 2.3 | Belgium |
| 140 | Impaired spreading of surfactant phospholipids in the lungs of newborn rats with pulmonary hypoplasia as a model of congenital diaphragmatic hernia induced by nitrofen | BIOCHIMICA ET BIOPHYSICA ACTA-MOLECULAR AND CELL BIOLOGY OF LIPIDS | 12 | 2001 | 200.0 | 2.2 | Japan |
| 141 | Antenatal dexamethasone administration increases fetal lung DNA synthesis and RNA and protein content in nitrofen-induced congenital diaphragmatic hernia in rats | PEDIATRIC RESEARCH | 12 | 2000 | 208.3 | 3.1 | Ireland |
| 142 | Intrauterine tracheal obstruction, a new treatment for congenital diaphragmatic hernia, decreases amniotic fluid sodium and chloride concentrations in the fetal lamb | ANNALS OF SURGERY | 12 | 1997 | 233.3 | 6.4 | Belgium |
| 143 | Increased intracellular levels of calcitonin gene-related peptide-like immunoreactivity in pulmonary endocrine cells in an experimental model of congenital diaphragmatic hernia | PEDIATRIC SURGERY INTERNATIONAL | 12 | 1996 | 241.7 | 1.6 | Ireland |
| 144 | Excessive Reversal of Epidermal Growth Factor Receptor and Ephrin Signaling Following Tracheal Occlusion in Rabbit Model of Congenital Diaphragmatic Hernia | MOLECULAR MEDICINE | 11 | 2016 | 81.8 | 6.4 | USA |
| 145 | Decreased Expression of GATA4 in the Diaphragm of Nitrofen-Induced Congenital Diaphragmatic Hernia | BIRTH DEFECTS RESEARCH PART B-DEVELOPMENTAL AND REPRODUCTIVE TOXICOLOGY | 11 | 2013 | 109.1 | 1.6 | Ireland |
| 146 | Expression of the Wilm's tumor gene WT1 during diaphragmatic development in the nitrofen model for congenital diaphragmatic hernia | PEDIATRIC SURGERY INTERNATIONAL | 11 | 2011 | 127.3 | 1.6 | Ireland |
| 147 | Hypoxic pulmonary vasoconstriction is impaired in rats with nitrofen-induced congenital diaphragmatic hernia | JOURNAL OF PEDIATRIC SURGERY | 11 | 1998 | 245.5 | 2.5 | USA |
| 148 | Pulmonary alveolar and vascular morphometry after gel plug occlusion of the trachea in a fetal rabbit model of CDH | INTERNATIONAL JOURNAL OF SURGERY | 11 | 2013 | 109.1 | 10.1 | USA |
| 149 | Low maternal vitamin A intake increases the incidence of teratogen induced congenital diaphragmatic hernia in mice | PEDIATRIC RESEARCH | 10 | 2022 | 30.0 | 3.1 | Canada |
| 150 | Pumpless arteriovenous extracorporeal membrane oxygenation: A novel mode of respiratory support in a lamb model of congenital diaphragmatic hernia | JOURNAL OF PEDIATRIC SURGERY | 10 | 2018 | 70.0 | 2.5 | USA |
| 151 | Antenatal BAY 41-2272 reduces pulmonary hypertension in the rabbit model of congenital diaphragmatic hernia | AMERICAN JOURNAL OF PHYSIOLOGY-LUNG CELLULAR AND MOLECULAR PHYSIOLOGY | 10 | 2016 | 90.0 | 3.5 | Belgium |
| 152 | Evidence for decreased lipofibroblast expression in hypoplastic rat lungs with congenital diaphragmatic hernia | PEDIATRIC SURGERY INTERNATIONAL | 10 | 2014 | 110.0 | 1.6 | Ireland |
| 153 | Increased contractility and impaired relaxation of the left pulmonary artery in a rabbit model of congenital diaphragmatic hernia | PEDIATRIC SURGERY INTERNATIONAL | 10 | 2013 | 120.0 | 1.6 | USA |
| 154 | Temporary fetal tracheal occlusion using a gel plug in a rabbit model of congenital diaphragmatic hernia | JOURNAL OF PEDIATRIC SURGERY | 10 | 2012 | 130.0 | 2.5 | USA |
| 155 | Dynamic tracheal occlusion improves lung morphometrics and function in the fetal lamb model of congenital diaphragmatic hernia | JOURNAL OF PEDIATRIC SURGERY | 10 | 2011 | 140.0 | 2.5 | USA |
| 156 | Tracheal innervation is abnormal in rats with experimental congenital diaphragmatic hernia | JOURNAL OF PEDIATRIC SURGERY | 10 | 2009 | 160.0 | 2.5 | Spain |
| 157 | Maternal administration of betamethasone inhibits proliferation induced by fetal tracheal occlusion in the nitrofen rat model for congenital diaphragmatic hernia: a placebo-controlled study | PEDIATRIC SURGERY INTERNATIONAL | 10 | 2008 | 170.0 | 1.6 | Belgium |
| 158 | Pulmonary elastin expression is decreased in the nitrofen-induced rat model of congenital diaphragmatic hernia | JOURNAL OF PEDIATRIC SURGERY | 10 | 2004 | 210.0 | 2.5 | USA |
| 159 | Effect of CGS 26303, an endothelin-converting enzyme-neutral endopeptidase inhibitor, on nitrofen-induced congenital diaphragmatic hernia in the rat | JOURNAL OF PEDIATRIC SURGERY | 10 | 2000 | 250.0 | 2.5 | Canada |
| 160 | Iatrogenic gastroschisis decreases pulmonary hypoplasia in an ovine congenital diaphragmatic hernia model | FETAL DIAGNOSIS AND THERAPY | 10 | 1995 | 300.0 | 1.6 | USA |
| 161 | The effect of tracheal occlusion on lung branching in the rat nitrofen CDH model | JOURNAL OF SURGICAL RESEARCH | 10 | 2008 | 170.0 | 1.7 | Canada |
| 162 | Phospholipid Profile of Amniotic Fluid in Ovine Model of Congenital Diaphragmatic Hernia (CDH): The Effect of Fetal Tracheal Occlusion | JOURNAL OF PROTEOME RESEARCH | 9 | 2015 | 111.1 | 3.6 | Italy |
| 163 | Disruption of copper-dependent signaling pathway in the nitrofen-induced congenital diaphragmatic hernia | PEDIATRIC SURGERY INTERNATIONAL | 9 | 2015 | 111.1 | 1.6 | Ireland |
| 164 | Amniotic lamellar body count and congenital diaphragmatic hernia in humans and in a rat model | PEDIATRIC RESEARCH | 9 | 2013 | 133.3 | 3.1 | Japan |
| 165 | Prenatal retinoic acid upregulates connexin 43 (Cx43) gene expression in pulmonary hypoplasia in the nitrofen-induced congenital diaphragmatic hernia rat model | JOURNAL OF PEDIATRIC SURGERY | 9 | 2012 | 144.4 | 2.5 | Ireland |
| 166 | Combining keratinocyte growth factor transfection into the airways and tracheal occlusion in a fetal sheep model of congenital diaphragmatic hernia | JOURNAL OF GENE MEDICINE | 9 | 2010 | 166.7 | 2.2 | France |
| 167 | Pulmonary epithelial cell differentiation in the nitrofen-induced congenital diaphragmatic hernia | JOURNAL OF PEDIATRIC SURGERY | 9 | 2007 | 200.0 | 2.5 | Portugal |
| 168 | Increased expression of ICAM-1 and VCAM-1 in the lung of nitrofen-induced congenital diaphragmatic hernia in rats | PEDIATRIC SURGERY INTERNATIONAL | 9 | 2003 | 244.4 | 1.6 | Ireland |
| 169 | Overexpression of miR-455-5p affects retinol (vitamin A) absorption by downregulating STRA6 in a nitrofen-induced CDH with lung hypoplasia rat model | PEDIATRIC PULMONOLOGY | 9 | 2020 | 55.6 | 2.3 | China |
| 170 | Transamniotic Stem Cell Therapy for Experimental Congenital Diaphragmatic Hernia: Structural, Transcriptional, and Cell Kinetics Analyses in the Nitrofen Model | FETAL DIAGNOSIS AND THERAPY | 8 | 2021 | 50.0 | 1.6 | USA |
| 171 | Resolving the heterogeneity of diaphragmatic mesenchyme: a novel mouse model of congenital diaphragmatic hernia | DISEASE MODELS & MECHANISMS | 8 | 2021 | 50.0 | 3.3 | UK |
| 172 | Intra-amniotic Sildenafil Treatment Modulates Vascular Smooth Muscle Cell Phenotype in the Nitrofen Model of Congenital Diaphragmatic Hernia | SCIENTIFIC REPORTS | 8 | 2018 | 87.5 | 3.9 | USA |
| 173 | Prenatal administration of neuropeptide bombesin promotes lung development in a rat model of nitrofen-induced congenital diaphragmatic hernia | JOURNAL OF PEDIATRIC SURGERY | 8 | 2014 | 137.5 | 2.5 | Japan |
| 174 | Nitrofen interferes with trophoblastic expression of retinol-binding protein and transthyretin during lung morphogenesis in the nitrofen-induced congenital diaphragmatic hernia model | PEDIATRIC SURGERY INTERNATIONAL | 8 | 2012 | 162.5 | 1.6 | Ireland |
| 175 | The Myenteric Plexus of the Esophagus is Abnormal in an Experimental Congenital Diaphragmatic Hernia Model | EUROPEAN JOURNAL OF PEDIATRIC SURGERY | 8 | 2009 | 200.0 | 1.4 | Spain |
| 176 | Pulmonary surfactant dysfunction in congenital diaphragmatic hernia: Experimental and clinical findings | BIOLOGY OF THE NEONATE | 8 | 1996 | 362.5 | 3 | Spain |
| 177 | Defective parasympathetic innervation is associated with airway branching abnormalities in experimental CDH | AMERICAN JOURNAL OF PHYSIOLOGY-LUNG CELLULAR AND MOLECULAR PHYSIOLOGY | 8 | 2015 | 125.0 | 3.5 | USA |
| 178 | Anti-inflammatory effects of antenatal administration of stem cell derived extracellular vesicles in the brain of rat fetuses with congenital diaphragmatic hernia | PEDIATRIC SURGERY INTERNATIONAL | 7 | 2023 | 28.6 | 1.6 | Canada |
| 179 | Validation of a Low-Cost Do-It-Yourself Model for Neonatal Thoracoscopic Congenital Diaphragmatic Hernia Repair | JOURNAL OF SURGICAL EDUCATION | 7 | 2018 | 100.0 | 2.1 | Portugal |
| 180 | Gata-6 expression is decreased in diaphragmatic and pulmonary mesenchyme of fetal rats with nitrofen-induced congenital diaphragmatic hernia | PEDIATRIC SURGERY INTERNATIONAL | 7 | 2018 | 100.0 | 1.6 | Ireland |
| 181 | Role of catalytic iron and oxidative stress in nitrofen-induced congenital diaphragmatic hernia and its amelioration by Saireito (TJ-114) | JOURNAL OF CLINICAL BIOCHEMISTRY AND NUTRITION | 7 | 2017 | 114.3 | 1.7 | Japan |
| 182 | Downregulation of KCNQ5 expression in the rat pulmonary vasculature of nitrofen-induced congenital diaphragmatic hernia | JOURNAL OF PEDIATRIC SURGERY | 7 | 2017 | 114.3 | 2.5 | Ireland |
| 183 | Fetal Cardiac Impairment in Nitrofen-Induced Congenital Diaphragmatic Hernia: Postmortem Microcomputed Tomography Imaging Study | FETAL AND PEDIATRIC PATHOLOGY | 7 | 2017 | 114.3 | 0.6 | Italy |
| 184 | Antenatal Saireito (TJ-114) Can Improve Pulmonary Hypoplasia and Pulmonary Vascular Remodeling in Nitrofen-Induced Congenital Diaphragmatic Hernia | PHYTOTHERAPY RESEARCH | 7 | 2016 | 128.6 | 6.3 | Japan |
| 185 | Combined antenatal therapy with retinoic acid and tracheal occlusion in a rat model of congenital diaphragmatic hernia | PEDIATRIC SURGERY INTERNATIONAL | 7 | 2016 | 128.6 | 1.6 | USA |
| 186 | Increased c-kit and stem cell factor expression in the pulmonary vasculature of nitrofen-induced congenital diaphragmatic hernia | JOURNAL OF PEDIATRIC SURGERY | 7 | 2016 | 128.6 | 2.5 | Ireland |
| 187 | The soluble guanylyl cyclase activator BAY 60-2770 potently relaxes the pulmonary artery on congenital diaphragmatic hernia rabbit model | PEDIATRIC SURGERY INTERNATIONAL | 7 | 2014 | 157.1 | 1.6 | Brazil |
| 188 | The effect of in vitro tracheal occlusion on branching morphogenesis in fetal lung explants from the rat nitrofen model of congenital diaphragmatic hernia | JOURNAL OF PEDIATRIC SURGERY | 7 | 2010 | 214.3 | 2.5 | Canada |
| 189 | Prenatal administration of retinoic acid upregulates connective tissue growth factor in the nitrofen CDH model | PEDIATRIC SURGERY INTERNATIONAL | 7 | 2011 | 200.0 | 1.6 | Ireland |
| 190 | Application of a postnatal prediction model of survival in CDH in the era of fetal therapy | JOURNAL OF MATERNAL-FETAL & NEONATAL MEDICINE | 7 | 2020 | 71.4 | 1.6 | Belgium |
| 191 | Prenatal treprostinil reduces the pulmonary hypertension phenotype in the rat model of congenital diaphragmatic hernia | EBIOMEDICINE | 6 | 2022 | 50.0 | 10.8 | Belgium |
| 192 | The fetal lamb model of congenital diaphragmatic hernia shows altered cerebral perfusion using contrast enhanced ultrasound | JOURNAL OF PEDIATRIC SURGERY | 6 | 2022 | 50.0 | 2.5 | USA |
| 193 | miR-200 family expression during normal and abnormal lung development due to congenital diaphragmatic hernia at the later embryonic stage in the nitrofen rat model | PEDIATRIC SURGERY INTERNATIONAL | 6 | 2020 | 83.3 | 1.6 | Canada |
| 194 | Upregulated EFNB2 and EPHB4 promotes lung development in a nitrofen-induced congenital diaphragmatic hernia rat model | INTERNATIONAL JOURNAL OF MOLECULAR MEDICINE | 6 | 2018 | 116.7 | 5.8 | China |
| 195 | Elastase and matrix metalloproteinase activities are associated with pulmonary vascular disease in the nitrofen rat model of congenital diaphragmatic hernia | JOURNAL OF PEDIATRIC SURGERY | 6 | 2017 | 133.3 | 2.5 | Canada |
| 196 | Imbalance of NFATc2 and KV1.5 Expression in Rat Pulmonary Vasculature of Nitrofen-Induced Congenital Diaphragmatic Hernia | EUROPEAN JOURNAL OF PEDIATRIC SURGERY | 6 | 2017 | 133.3 | 1.4 | Ireland |
| 197 | Antenatal retinoic acid administration increases trophoblastic retinol-binding protein dependent retinol transport in the nitrofen model of congenital diaphragmatic hernia | PEDIATRIC RESEARCH | 6 | 2016 | 150.0 | 3.1 | Ireland |
| 198 | Mesenchymal expression of the FRAS1/FREM2 gene unit is decreased in the developing fetal diaphragm of nitrofen-induced congenital diaphragmatic hernia | PEDIATRIC SURGERY INTERNATIONAL | 6 | 2016 | 150.0 | 1.6 | Ireland |
| 199 | Upregulation of S1P1 and Rac1 receptors in the pulmonary vasculature of nitrofen-induced congenital diaphragmatic hernia | PEDIATRIC SURGERY INTERNATIONAL | 6 | 2016 | 150.0 | 1.6 | Ireland |
| 200 | The Effect of Transplacental Administration of Glucagon-Like Peptide-1 on Fetal Lung Development in the Rabbit Model of Congenital Diaphragmatic Hernia | FETAL DIAGNOSIS AND THERAPY | 6 | 2016 | 150.0 | 1.6 | Belgium |
| 201 | Increased pulmonary vascular expression of receptor for advanced glycation end products (RAGE) in experimental congenital diaphragmatic hernia | JOURNAL OF PEDIATRIC SURGERY | 6 | 2015 | 166.7 | 2.5 | Ireland |
| 202 | Increased pulmonary vascular expression of Kruppel-like factor 5 and activated survivin in experimental congenital diaphragmatic hernia | PEDIATRIC SURGERY INTERNATIONAL | 6 | 2014 | 183.3 | 1.6 | Ireland |
| 203 | Aberrant pulmonary lymphatic development in the nitrofen mouse model of congenital diaphragmatic hernia | JOURNAL OF PEDIATRIC SURGERY | 6 | 2013 | 200.0 | 2.5 | USA |
| 204 | Decreased pulmonary c-Cbl expression and tyrosine phosphorylation in the nitrofen-induced rat model of congenital diaphragmatic hernia | PEDIATRIC SURGERY INTERNATIONAL | 6 | 2013 | 200.0 | 1.6 | Ireland |
| 205 | Pax3 gene expression is not altered during diaphragmatic development in nitrofen-induced congenital diaphragmatic hernia | JOURNAL OF PEDIATRIC SURGERY | 6 | 2012 | 216.7 | 2.5 | Ireland |
| 206 | ANG-1 TIE-2 and BMPR Signalling Defects Are Not Seen in the Nitrofen Model of Pulmonary Hypertension and Congenital Diaphragmatic Hernia | PLOS ONE | 6 | 2012 | 216.7 | 2.6 | UK |
| 207 | NKCC-1 and ENaC are down-regulated in nitrofen-induced hypoplastic lungs with congenital diaphragmatic hernia | PEDIATRIC SURGERY INTERNATIONAL | 6 | 2008 | 283.3 | 1.6 | Sweden |
| 208 | Effect of prenatal glucocorticoids and postnatal nitric oxide inhalation on survival of newborn rats with nitrofen-induced congenital diaphragmatic hernia | JOURNAL OF PEDIATRIC SURGERY | 6 | 2002 | 383.3 | 2.5 | Germany |
| 209 | Effect of immediate versus slow intrauterine reduction of congenital diaphragmatic hernia on lung development in the sheep: A morphometric analysis of term pulmonary structure and maturity | PEDIATRIC PULMONOLOGY | 6 | 2000 | 416.7 | 2.3 | Australia |
| 210 | Early functional analysis on the pulmonary hemodynamic effects of Transamniotic Stem Cell Therapy (TRASCET) in the nitrofen model of congenital diaphragmatic hernia | JOURNAL OF PEDIATRIC SURGERY | 5 | 2023 | 40.0 | 2.5 | USA |
| 211 | An Implantable Electronic Device for Monitoring Fetal Lung Pressure in a Lamb Model of Congenital Diaphragmatic Hernia | IEEE TRANSACTIONS ON INSTRUMENTATION AND MEASUREMENT | 5 | 2022 | 60.0 | 5.9 | Italy |
| 212 | Expression of Nitric Oxide Synthase Interacting Protein (NOSIP) is Decreased in the Pulmonary Vasculature of Nitrofen-Induced Congenital Diaphragmatic Hernia | EUROPEAN JOURNAL OF PEDIATRIC SURGERY | 5 | 2019 | 120.0 | 1.4 | Ireland |
| 213 | Suppressed Erythropoietin Expression in a Nitrofen-Induced Congenital Diaphragmatic Hernia | PEDIATRIC PULMONOLOGY | 5 | 2017 | 160.0 | 2.3 | Japan |
| 214 | Increased pulmonary RhoA expression in the nitrofen-induced congenital diaphragmatic hernia rat model | JOURNAL OF PEDIATRIC SURGERY | 5 | 2015 | 200.0 | 2.5 | Japan |
| 215 | Lower NPAS3 expression during the later stages of abnormal lung development in rat congenital diaphragmatic hernia | PEDIATRIC SURGERY INTERNATIONAL | 5 | 2015 | 200.0 | 1.6 | Canada |
| 216 | Early neonatal echocardiographic findings in an experimental rabbit model of congenital diaphragmatic hernia | BRAZILIAN JOURNAL OF MEDICAL AND BIOLOGICAL RESEARCH | 5 | 2015 | 200.0 | 1.5 | Brazil |
| 217 | Full thickness abdominal wall defect in growing rats as a model for congenital diaphragmatic hernia prosthetic repair | JOURNAL OF PEDIATRIC SURGERY | 5 | 2014 | 220.0 | 2.5 | Belgium |
| 218 | Nitrofen increases total retinol levels in placenta during lung morphogenesis in the nitrofen model of congenital diaphragmatic hernia | PEDIATRIC SURGERY INTERNATIONAL | 5 | 2014 | 220.0 | 1.6 | Ireland |
| 219 | Upregulation of serotonin-receptor-2a and serotonin transporter expression in the pulmonary vasculature of nitrofen-induced congenital diaphragmatic hernia | JOURNAL OF PEDIATRIC SURGERY | 5 | 2014 | 220.0 | 2.5 | Ireland |
| 220 | Downregulation of FGFRL1 Contributes to the Development of the Diaphragmatic Defect in the Nitrofen Model of Congenital Diaphragmatic Hernia | EUROPEAN JOURNAL OF PEDIATRIC SURGERY | 5 | 2011 | 280.0 | 1.4 | Ireland |
| 221 | Expression and Function of Phosphodiesterases in Nitrofen-Induced Congenital Diaphragmatic Hernia in Rats | PEDIATRIC PULMONOLOGY | 5 | 2010 | 300.0 | 2.3 | Canada |
| 222 | T2 quantifications of lungs in the fetal lamb with experimentally-induced congenital diaphragmatic hernia | FETAL DIAGNOSIS AND THERAPY | 5 | 2007 | 360.0 | 1.6 | Belgium |
| 223 | Kidney development in the nitrofen-induced pulmonary hypolasia and congenital diaphragmatic hernia in rats | JOURNAL OF PEDIATRIC SURGERY | 5 | 2007 | 360.0 | 2.5 | Ireland |
| 224 | Antenatal vitamin A decreases ventilation-induced lung injury in the lamb model of congenital diaphragmatic hernia | ASIAN JOURNAL OF SURGERY | 5 | 2006 | 380.0 | 3.8 | USA |
| 225 | Can maternal vitamin E supplementation prevent lung hypoplasia in the nitrofen-induced rat model of congenital diaphragmatic hernia? | PEDIATRIC RESEARCH | 5 | 2005 | 400.0 | 3.1 | USA |
| 226 | Cardiac gene expression and synthesis of atrial natriuretic peptide in the nitrofen model of congenital diaphragmatic hernia in rats: Effect of prenatal dexamethazone treatment | JOURNAL OF PEDIATRIC SURGERY | 5 | 2001 | 480.0 | 2.5 | Ireland |
| 227 | Tracheal ligation and mechanical ventilation do not improve the antioxidant enzyme status in the lamb model of congenital diaphragmatic hernia | JOURNAL OF PEDIATRIC SURGERY | 5 | 1999 | 520.0 | 2.5 | USA |
| 228 | Effect of prenatal tetrandrine therapy on pulmonary vascular structural remodeling in the nitrofen-induced CDH rat model | CHINESE MEDICAL JOURNAL | 5 | 2000 | 500.0 | 7.3 | China |
| 229 | Characterization of the congenital diaphragmatic hernia model in C57BL/6J fetal mice: a step toward lineage tracing experiments | PEDIATRIC SURGERY INTERNATIONAL | 4 | 2023 | 50.0 | 1.6 | Canada |
| 230 | Brain development is altered in rabbit fetuses with congenital diaphragmatic hernia | PRENATAL DIAGNOSIS | 4 | 2023 | 50.0 | 2.7 | Belgium |
| 231 | Liver pathological alterations in fetal rabbit model of congenital diaphragmatic hernia | CONGENITAL ANOMALIES | 4 | 2022 | 75.0 | 1.6 | Italy |
| 232 | Heterogeneous Response in Rabbit Fetal Diaphragmatic Hernia Lungs After Tracheal Occlusion | JOURNAL OF SURGICAL RESEARCH | 4 | 2020 | 125.0 | 1.7 | USA |
| 233 | Lung function and pulmonary artery blood flow following prenatal maternal retinoic acid and imatinib in the nitrofen model of congenital diaphragmatic hernia | JOURNAL OF PEDIATRIC SURGERY | 4 | 2018 | 175.0 | 2.5 | USA |
| 234 | Follistatin-like 1 expression is decreased in the alveolar epithelium of hypoplastic rat lungs with nitrofen-induced congenital diaphragmatic hernia | JOURNAL OF PEDIATRIC SURGERY | 4 | 2017 | 200.0 | 2.5 | Ireland |
| 235 | Prenatal administration of all-trans retinoic acid upregulates leptin signaling in hypoplastic rat lungs with experimental congenital diaphragmatic hernia | PEDIATRIC SURGERY INTERNATIONAL | 4 | 2014 | 275.0 | 1.6 | Ireland |
| 236 | Increased uptake of dietary retinoids at the maternal-fetal barrier in the nitrofen model of congenital diaphragmatic hernia | JOURNAL OF PEDIATRIC SURGERY | 4 | 2014 | 275.0 | 2.5 | Ireland |
| 237 | Prenatal administration of retinoic acid increases the trophoblastic insulin-like growth factor 2 protein expression in the nitrofen model of congenital diaphragmatic hernia | PEDIATRIC SURGERY INTERNATIONAL | 4 | 2014 | 275.0 | 1.6 | Ireland |
| 238 | The RNA-binding protein quaking is upregulated in nitrofen-induced congenital diaphragmatic hernia lungs at the end of gestation | PEDIATRIC SURGERY INTERNATIONAL | 3 | 2024 | 33.3 | 1.6 | Canada |
| 239 | The effect of tracheal occlusion in congenital diaphragmatic hernia in the nitrofen rat lung explant model | PEDIATRIC SURGERY INTERNATIONAL | 3 | 2023 | 66.7 | 1.6 | Canada |
| 240 | Identification of SOX2 Interacting Proteins in the Developing Mouse Lung With Potential Implications for Congenital Diaphragmatic Hernia | FRONTIERS IN PEDIATRICS | 3 | 2022 | 100.0 | 2 | Netherlands |
| 241 | Neonatal rodent ventilation and clinical correlation in congenital diaphragmatic hernia | PEDIATRIC PULMONOLOGY | 3 | 2022 | 100.0 | 2.3 | USA |
| 242 | Yes-associated protein is dysregulated during nitrofen-induced hypoplastic lung development due to congenital diaphragmatic hernia | PEDIATRIC SURGERY INTERNATIONAL | 3 | 2022 | 100.0 | 1.6 | Canada |
| 243 | Vitamin D improves pulmonary function in a rat model for congenital diaphragmatic hernia | ARCHIVES OF BIOCHEMISTRY AND BIOPHYSICS | 3 | 2021 | 133.3 | 3 | Japan |
| 244 | Intra-Amniotic Sildenafil Treatment Promotes Lung Growth and Attenuates Vascular Remodeling in an Experimental Model of Congenital Diaphragmatic Hernia | FETAL DIAGNOSIS AND THERAPY | 3 | 2020 | 166.7 | 1.6 | USA |
| 245 | Tandem mass tag (TMT) proteomic analysis of fetal lungs revealed differential expression of tight junction proteins in a rat model of congenital diaphragmatic hernia | BIOMEDICINE & PHARMACOTHERAPY | 3 | 2020 | 166.7 | 7.5 | China |
| 246 | Vascular and ventilatory mechanical responses in three different stages of pulmonary development in the rabbit model of congenital diaphragmatic hernia | ACTA CIRURGICA BRASILEIRA | 3 | 2018 | 233.3 | 1.3 | Brazil |
| 247 | Increased CaSR and TRPC6 pulmonary vascular expression in the nitrofen-induced model of congenital diaphragmatic hernia | PEDIATRIC SURGERY INTERNATIONAL | 3 | 2018 | 233.3 | 1.6 | Ireland |
| 248 | Downregulated Elastin Microfibril Interfacer 1 Expression in the Pulmonary Vasculature of Experimental Congenital Diaphragmatic Hernia | EUROPEAN JOURNAL OF PEDIATRIC SURGERY | 3 | 2018 | 233.3 | 1.4 | Ireland |
| 249 | Fibrillin-1 Expression Is Decreased in the Diaphragmatic Muscle Connective Tissue of Nitrofen-Induced Congenital Diaphragmatic Hernia | EUROPEAN JOURNAL OF PEDIATRIC SURGERY | 3 | 2017 | 266.7 | 1.4 | Ireland |
| 250 | Decreased Endoglin expression in the pulmonary vasculature of nitrofen-induced congenital diaphragmatic hernia rat model | PEDIATRIC SURGERY INTERNATIONAL | 3 | 2017 | 266.7 | 1.6 | Ireland |
| 251 | Decreased Desmin expression in the developing diaphragm of the nitrofen-induced congenital diaphragmatic hernia rat model | PEDIATRIC SURGERY INTERNATIONAL | 3 | 2016 | 300.0 | 1.6 | Ireland |
| 252 | Expression of hepatic lipid droplets is decreased in the nitrofen model of congenital diaphragmatic hernia | PEDIATRIC SURGERY INTERNATIONAL | 3 | 2016 | 300.0 | 1.6 | Ireland |
| 253 | Gene Expression of FRAS1-Related Extracellular Matrix 1 Is Decreased in Nitrofen-Induced Congenital Diaphragmatic Hernia | EUROPEAN JOURNAL OF PEDIATRIC SURGERY | 3 | 2016 | 300.0 | 1.4 | Ireland |
| 254 | Enteric Nervous System and Esophageal-Gastrointestinal Motility in Experimental Congenital Diaphragmatic Hernia | EUROPEAN JOURNAL OF PEDIATRIC SURGERY | 3 | 2014 | 366.7 | 1.4 | Spain |
| 255 | Increased trophoblastic apoptosis mediated by neutrophil gelatinase-associated lipocalin (NGAL) activation in the nitrofen model of congenital diaphragmatic hernia | PEDIATRIC SURGERY INTERNATIONAL | 3 | 2013 | 400.0 | 1.6 | Ireland |
| 256 | Intraluminal tracheal occlusion using a modified 8-mm Z-stent in a sheep model of left-sided congenital diaphragmatic hernia | JOURNAL OF MATERNAL-FETAL & NEONATAL MEDICINE | 3 | 2012 | 433.3 | 1.6 | Venezuela |
| 257 | COUP-TFII Gene Expression is Upregulated in Embryonic Pleuroperitoneal Folds in the Nitrofen-Induced Congenital Diaphragmatic Hernia Rat Model | EUROPEAN JOURNAL OF PEDIATRIC SURGERY | 3 | 2012 | 433.3 | 1.4 | Ireland |
| 258 | Abnormal development of the enteric nervous system in rat embryos and fetuses with congenital diaphragmatic hernia | PEDIATRIC SURGERY INTERNATIONAL | 3 | 2011 | 466.7 | 1.6 | Spain |
| 259 | Is there a role for antioxidants in prevention of pulmonary hypoplasia in nitrofen-induced rat model of congenital diaphragmatic hernia? | PEDIATRIC SURGERY INTERNATIONAL | 3 | 2010 | 500.0 | 1.6 | Turkey |
| 260 | Antenatal dexamethasone administration inhibits smooth-muscle-cell DNA synthesis in pulmonary-arterial media in nitrofen-induced congenital diaphragmatic hernia in rats | PEDIATRIC SURGERY INTERNATIONAL | 3 | 2000 | 833.3 | 1.6 | Ireland |
| 261 | Effect of insulin-like growth factors on lung development in a nitrofen-induced CDH rat model | PEDIATRIC SURGERY INTERNATIONAL | 3 | 2011 | 466.7 | 1.6 | Japan |
| 262 | The metabolic and lipidomic profiling of the effects of tracheal occlusion in a rabbit model of congenital diaphragmatic hernia | JOURNAL OF PEDIATRIC SURGERY | 2 | 2023 | 100.0 | 2.5 | Canada |
| 263 | Fetal lung hypoxia and energetic cell failure in the nitrofen-induced congenital diaphragmatic hernia rat model | PEDIATRIC SURGERY INTERNATIONAL | 2 | 2023 | 100.0 | 1.6 | USA |
| 264 | Development and internal validation of a prediction model to predict survival for congenital diaphragmatic hernia in the early postnatal period | JOURNAL OF MATERNAL-FETAL & NEONATAL MEDICINE | 2 | 2022 | 150.0 | 1.6 | China |
| 265 | Pbx1, Meis1, and Runx1 Expression Is Decreased in the Diaphragmatic and Pulmonary Mesenchyme of Rats with Nitrofen-Induced Congenital Diaphragmatic Hernia | EUROPEAN JOURNAL OF PEDIATRIC SURGERY | 2 | 2021 | 200.0 | 1.4 | Ireland |
| 266 | Anti-oxidants correct disturbance of redox enzymes in the hearts of rat fetuses with congenital diaphragmatic hernia | PEDIATRIC SURGERY INTERNATIONAL | 2 | 2018 | 350.0 | 1.6 | Spain |
| 267 | Protective effect of baicalin on fetal lung development in a rabbit model of congenital diaphragmatic hernia | EXPERIMENTAL AND THERAPEUTIC MEDICINE | 2 | 2018 | 350.0 | 2.3 | China |
| 268 | Evaluation of alveolar epithelial cells in the sheep model of congenital diaphragmatic hernia: Type 1 alveolar epithelial cells and histopathological image analysis | JOURNAL OF PEDIATRIC SURGERY | 2 | 2017 | 400.0 | 2.5 | Japan |
| 269 | Downregulation of Forkhead box F1 gene expression in the pulmonary vasculature of nitrofen-induced congenital diaphragmatic hernia | PEDIATRIC SURGERY INTERNATIONAL | 2 | 2016 | 450.0 | 1.6 | Ireland |
| 270 | Decreased expression of hepatocyte growth factor in the nitrofen model of congenital diaphragmatic hernia | PEDIATRIC SURGERY INTERNATIONAL | 2 | 2016 | 450.0 | 1.6 | Ireland |
| 271 | Tracheal occlusion and ventilation changes the nitric oxide pathway in congenital diaphragmatic hernia model | JOURNAL OF SURGICAL RESEARCH | 2 | 2016 | 450.0 | 1.7 | Brazil |
| 272 | Fetal Skin Fibroblasts: A Cell Model for Studying the Retinoid Pathway in Congenital Diaphragmatic Hernia | BIRTH DEFECTS RESEARCH PART A-CLINICAL AND MOLECULAR TERATOLOGY | 2 | 2010 | 750.0 | 1.6 | France |
| 273 | The impact of iatrogenic gastroschisis on pulmonary maturation in the fetal rabbit models of congenital diaphragmatic hernia | PEDIATRIC SURGERY INTERNATIONAL | 2 | 2009 | 800.0 | 1.6 | China |
| 274 | Myocardium expression of connexin 43, SERCA2a, and myosin heavy chain isoforms are preserved in nitrofen-induced congenital diaphragmatic hernia rat model | JOURNAL OF PEDIATRIC SURGERY | 2 | 2006 | 950.0 | 2.5 | Portugal |
| 275 | The adrenal cortex in experimental congenital diaphragmatic hernia | JOURNAL OF PEDIATRIC SURGERY | 2 | 2003 | 1100.0 | 2.5 | Spain |
| 276 | Sex-specific differences in the severity of pulmonary hypoplasia in experimental congenital diaphragmatic hernia and implications for extracellular vesicle-based therapy | PEDIATRIC SURGERY INTERNATIONAL | 1 | 2024 | 100.0 | 1.6 | Canada |
| 277 | Experimental congenital diaphragmatic hernia features an alteration of DNA sensing targets cGAS and STING | PEDIATRIC RESEARCH | 1 | 2024 | 100.0 | 3.1 | Germany |
| 278 | Development of a 3D-printed neonatal congenital diaphragmatic hernia model and standardisation of intra-operative measurement | PEDIATRIC SURGERY INTERNATIONAL | 1 | 2023 | 200.0 | 1.6 | UK |
| 279 | Prenatal treprostinil improves pulmonary arteriolar hypermuscularization in the rabbit model of congenital diaphragmatic hernia | BIOMEDICINE & PHARMACOTHERAPY | 1 | 2024 | 100.0 | 7.5 | Belgium |
| 280 | A novel congenital diaphragmatic hernia prediction model for Chinese subjects: A multicenter cohort investigation | HELIYON | 1 | 2023 | 200.0 | 3.6 | China |
| 281 | The myocardial capillary network is altered in congenital diaphragmatic hernia in the fetal rabbit model | BRAZILIAN JOURNAL OF MEDICAL AND BIOLOGICAL RESEARCH | 1 | 2023 | 200.0 | 1.5 | Brazil |
| 282 | Congenital diaphragmatic hernia: phosphodiesterase-5 and Arginase inhibitors prevent pulmonary vascular hypoplasia in rat lungs | PEDIATRIC RESEARCH | 1 | 2024 | 100.0 | 3.1 | Chile |
| 283 | Stereological Lung Parameters After Fetoscopic Abdominal Decompression of Congenital Diaphragmatic Hernia in an Ovine Model: A Pilot Study | JOURNAL OF LAPAROENDOSCOPIC & ADVANCED SURGICAL TECHNIQUES | 1 | 2021 | 400.0 | 1.1 | Germany |
| 284 | Involvement of YAP and LATS1 in lung development in a rat model of nitrofen-induced congenital diaphragmatic hernia | TROPICAL JOURNAL OF PHARMACEUTICAL RESEARCH | 1 | 2020 | 500.0 | 0.6 | China |
| 285 | Ephrin-B1,-B2, and-B4 Expression is Decreased in Developing Diaphragms and Lungs of Fetal Rats with Nitrofen-Induced Congenital Diaphragmatic Hernia | EUROPEAN JOURNAL OF PEDIATRIC SURGERY | 1 | 2019 | 600.0 | 1.4 | Ireland |
| 286 | Surgical technique for developing a rabbit model of congenital diaphragmatic hernia and tracheal occlusion | METHODSX | 1 | 2019 | 600.0 | 1.9 | Canada |
| 287 | Down-regulation of N-deacetylase-N-sulfotransferase-1 signaling in the developing diaphragmatic vasculature of nitrofen-induced congenital diaphragmatic hernia | JOURNAL OF PEDIATRIC SURGERY | 1 | 2017 | 800.0 | 2.5 | Ireland |
| 288 | Congenital diaphragmatic hernia: endotracheal fluid phospholipidic profile following tracheal occlusion in an experimental model | JOURNAL OF PERINATAL MEDICINE | 1 | 2017 | 800.0 | 1.4 | Italy |
| 289 | Expression of Prx1 and Tcf4 is decreased in the diaphragmatic muscle connective tissue of nitrofen-induced congenital diaphragmatic hernia | JOURNAL OF PEDIATRIC SURGERY | 1 | 2016 | 900.0 | 2.5 | Ireland |
| 290 | Decreased expression of monocarboxylate transporter 1 and 4 in the branching airway epithelium of nitrofen-induced congenital diaphragmatic hernia | JOURNAL OF PEDIATRIC SURGERY | 1 | 2016 | 900.0 | 2.5 | Ireland |
| 291 | Bochdalek Congenital Diaphragmatic Hernia in an Adult Sheep | ANATOMIA HISTOLOGIA EMBRYOLOGIA | 1 | 2016 | 900.0 | 1 | USA |
| 292 | Decidual β-carotene-15,15′-oxygenase-1 and 2 (BCMO1,2) expression is increased in nitrofen model of congenital diaphragmatic hernia | PEDIATRIC SURGERY INTERNATIONAL | 1 | 2015 | 1000.0 | 1.6 | Ireland |
| 293 | Epimorphin expression in a rat model of pulmonary hypoplasia associated with congenital diaphragmatic hernia | PEDIATRIC SURGERY INTERNATIONAL | 1 | 2014 | 1100.0 | 1.6 | Turkey |
| 294 | Myogenin gene expression is not altered in the developing diaphragm of nitrofen-induced congenital diaphragmatic hernia | PEDIATRIC SURGERY INTERNATIONAL | 1 | 2014 | 1100.0 | 1.6 | Ireland |
| 295 | Comparative Transcriptome Analysis of Human and Mouse Canalicular Lungs in Fetal Diaphragmatic Hernia | JOURNAL OF PEDIATRIC SURGERY | 0 | 2024 | NA | 2.5 | USA |
| 296 | Endothelial-to-Mesenchymal Transition in Human and Murine Models of Congenital Diaphragmatic Hernia | NEONATOLOGY | 0 | 2024 | NA | 3 | USA |
| 297 | Intact cord resuscitation in newborns with congenital diaphragmatic hernia: insights from a lamb model | FRONTIERS IN PEDIATRICS | 0 | 2023 | NA | 2 | France |
| 298 | Upregulated TSP1 and CD47 expression in the lung in nitrofen-induced congenital diaphragmatic hernia | PEDIATRICS INTERNATIONAL | 0 | 2023 | NA | 0.9 | Japan |
| 299 | Comparison of CDHSG model and PCO2 in predicting mortality risk in patients with congenital diaphragmatic hernia | CONGENITAL ANOMALIES | 0 | 2022 | NA | 1.6 | Turkey |
| 300 | Prenatal Molecular Hydrogen Administration Ameliorates Several Findings in Nitrofen-Induced Congenital Diaphragmatic Hernia | INTERNATIONAL JOURNAL OF MOLECULAR SCIENCES | 0 | 2021 | NA | 4.9 | Japan |
| 301 | Effect of antenatal tetramethylpyrazine on lung development and YAP expression in rat model of experimental congenital diaphragmatic hernia | INTERNATIONAL JOURNAL OF CLINICAL AND EXPERIMENTAL PATHOLOGY | 0 | 2020 | NA | 0.9 | China |
| 302 | Expression of dispatched RND transporter family member 1 is decreased in the diaphragmatic and pulmonary mesenchyme of nitrofen-induced congenital diaphragmatic hernia | PEDIATRIC SURGERY INTERNATIONAL | 0 | 2019 | NA | 1.6 | Ireland |

NA: Not applicable.
